# Quantitative Approach to Quality Review of Prenatal Ultrasound Examinations: Incomplete Detailed Fetal Anatomy Exams

**DOI:** 10.3390/jcm14103356

**Published:** 2025-05-12

**Authors:** C. Andrew Combs, Olaide Ashimi Balogun, Jeroen Vanderhoeven, Sushma Amara

**Affiliations:** 1 Pediatrix Center for Research, Education, Quality & Safety, Sunrise, FL 33323, USA; 2 Obstetrix Maternal-Fetal Medicine Specialists, Houston, TX 77054, USA; olaide.ashimibalogun@pediatrix.com; 3 Maternal-Fetal Medicine Specialists of Puget Sound, Seattle, WA 98104, USA; jeroen.vanderhoeven@pediatrix.com; 4 Eastside Maternal-Fetal Medicine, Bellevue, WA 98004, USA; sushma.amara@pediatrix.com

**Keywords:** birth defects, cesarean delivery, congenital anomalies, diagnostic error, maternal age, maternal obesity, prenatal diagnosis

## Abstract

**Background/Objectives:** It is challenging to obtain all the required views for a fetal anatomy ultrasound examination, so exams are often incomplete. Our objective was to develop and demonstrate quantitative methods to assess the overall rate of incomplete exams for an ultrasound practice and for individual examiners. **Methods:** We performed a retrospective quality review of all detailed fetal anatomy exams at seven maternal–fetal medicine practices in 2024 with singleton pregnancies and cardiac activity present. The exams were considered incomplete if any of the 36 required anatomy views were reported as inadequate. The analysis focused on exams at a gestational age (GA) of 18.0 to 23.9 weeks. The rates of incomplete exams were tabulated across practices and for individual sonographers and physicians. Multivariable logistic regression was used to adjust for known covariates. **Results:** In total, 15,723 detailed fetal anatomy exams were performed at 18.0–23.9 weeks of gestation. Incomplete exams were significantly more common with maternal obesity, prior cesarean, maternal age < 35 years and GA < 19 weeks. There were significant between-practice differences in the rate of incomplete exams, varying from 1% to 53%. Incomplete exams had a median of four inadequate views (interquartile range 2–7). Practices also varied significantly in the rate of missing measurements for nuchal fold (0 to 9%) and nose bone length (11–100%). There were significant between-individual differences in the rate of incomplete exams. The tabulation of specific views showed some individuals with very high rates of inadequate views of certain elements. **Conclusions:** For some practices, there is a need for practice-wide quality improvement to increase the rate of measurement of the nuchal fold and nose bone. For selected individuals, the tabulation of which anatomy elements were inadequate can identify areas for targeted education or mentorship. We suggest strategies and software enhancements that may reduce the rate of incomplete exams. Sample data and statistical analysis scripts are provided for those who wish to adopt these methods to review their own data.

## 1. Introduction

The prenatal detection of congenital anomalies is an important goal of obstetrical ultrasound examination. The diagnosis of an anomaly has profound implications for counseling about prognosis and for guiding decisions about prenatal care, fetal surveillance and delivery timing, location and method.

To maximize prenatal detection of anomalies, standards from the American Institute of Ultrasound in Medicine [1,2] specify 52 required views for detailed fetal anatomy ultrasound examination in the second or third trimester of pregnancy. It is often challenging to obtain all the required views. Prior studies have reported that anatomy exams are incomplete in 4–70% of patients [3,4,5,6,7,8,9,10,11]. There are several reasons why skilled examiners might be unable to obtain a complete examination, including unfavorable fetal position, early gestational age, maternal obesity, maternal abdominal wall scarring (e.g., prior cesarean delivery) or insufficient time being allotted to complete the exam [3,4,6,7,9,10,12,13,14,15,16,17]. Moreover, examiners may have variable skill-levels in the evaluation of some structures.

Various professional organizations recommend systematic quality reviews to assure the accuracy of obstetrical ultrasound diagnoses [18,19,20,21]. Because ultrasound is an imaging modality, quality reviews must ultimately involve an examination of images. However, image auditing is time consuming, labor intensive and somewhat subjective. We recently presented quantitative methods for the quality review of fetal biometric measurements [22] and the review of the accuracy of fetal weight estimates [23]. We showed how image audits could be focused on sonographers or physicians whose measurements varied from the measurements of their peers, thereby saving the time and expense of auditing those whose measurements fell within the expected range. If examiners vary in the accuracy of their biometric measurements, it seems likely that they might also vary in their rate of obtaining complete fetal anatomy exams.

The objective of the present study was to develop and demonstrate quantitative methods to assess the rate of incomplete fetal anatomy exams for an ultrasound practice overall and for individual sonographers and physicians within a practice.

## 2. Materials and Methods

### 2.1. Study Design and Setting

A retrospective quality review was undertaken for the cohort of ultrasound exams performed from January through December 2024 at seven referral-based maternal–fetal medicine (MFM) practices affiliated with Pediatrix Medical Group (Sunrise, FL, USA). During the study period, the practices used GE Voluson (GE Healthcare, Wauwatosa, WI, USA) and Philips Affiniti (Philips Ultrasound LLC, Bothell, WA, USA) ultrasound machines with variable-frequency curvilinear transducers (2 to 9 MHz as needed to optimize imaging). The practices used GE Viewpoint software (Version 6) to generate reports and store images and metadata. The sonographers were certified by the American Registry for Diagnostic Medical Sonography. The interpreting physicians were board-certified or board-eligible MFM specialists.

Before we started the study, the Western Institutional Review Board (WIRB) determined that the quality review of existing ultrasound data was not considered human subject research, and therefore determined that the study was exempt.

### 2.2. Data Extraction and Inclusion and Exclusion Criteria

Using the Viewpoint query tool, we extracted data for all ultrasound exams meeting these eligibility criteria: singleton pregnancy; detailed fetal anatomy exam using Current Procedural Terminology code 76811 during 2024; fetal cardiac activity present; exam finalized with signed report in Viewpoint. The data were exported from Viewpoint to a comma-separated value file (.CSV format), which was converted into a spreadsheet file (.XLSX format) and imported into Stata statistical software (version BE/18.0, StataCorp, College Station, TX, USA) for analysis.

The extracted data included the required elements of the fetal anatomy survey, an exam identification number, a person identification number, the indications for the exam, the exam date, the patient’s date of birth (DOB), the estimated due date (EDD) based on the best obstetrical estimate (typically following consensus guidelines [24]), the sonographer’s name and the reading physician’s name. The person identification number was used to identify the first exam for each person; for persons with more than one eligible exam, we kept only the first and excluded any subsequent exam. The EDD was used to calculate gestational age (GA) on the day of the exam. GA was expressed as a decimal fraction rounded to 1 significant digit (e.g., 18 weeks + 4 days was expressed as 18.6 weeks and 23 weeks + 6 days is expressed as 23.9 weeks). The DOB was used to calculate maternal age. Advanced maternal age was defined as an age ≥ 35 years on the EDD. After these calculations and exclusions, the person number, DOB and EDD were deleted to protect patient anonymity. From the indications, we determined whether there was notation of prior cesarean and whether there was notation of maternal obesity, which was categorized by maternal body mass index (BMI).

### 2.3. Definitions of Inadequate Views and Incomplete Examination

For each element of the anatomy survey, a text description of the findings was entered in Viewpoint by the sonographer or reading physician when creating the ultrasound report. To simplify data entry, Viewpoint has an option to select “all normal”, which initially categorizes every item as normal. Then, each entry can be modified, if needed, by selecting a different word or phrase from a pull-down list. For our analysis, we recoded the entries to fit into one of three categories:

- Normal if the item was entered as normal or previously documented, seen or visualized;
- Abnormal if it was entered as abnormal, a soft marker was seen or details were provided;
- Inadequate if it was entered as suboptimal, not seen or not examined, or if left blank.

The fetal hands, feet and lungs had separate entries in Viewpoint for the right and left side. These were each combined into a single element coded as normal if both sides were normal, abnormal if either side was abnormal, and inadequate otherwise. Superior and inferior vena cava views are separate entries in Viewpoint and were similarly combined into a single element.

We defined a fetal anatomy survey as incomplete if there were any items coded as inadequate among the 36 specific elements of fetal anatomy listed in the AIUM Detailed 2nd Trimester OB imaging checklist [2]. The checklist also contains 16 other items that we did not consider in the definition of a complete fetal anatomy survey: 2 items of maternal anatomy (uterus-and-cervix, adnexal structures); 6 placental or fetal findings that are not fetal anatomy per se (placental location, placental cord insertion, fetal number and presentation, amniotic fluid assessment, fetal heart rate, placental vasculature if accessory lobe); 3 fetal anatomy findings for which there is not a corresponding field in Viewpoint (position and architecture of legs and arms, shape and curvature of spine, integrity of spine and overlying tissue); and 5 fetal measurements (head circumference [HC] or biparietal diameter [BPD], abdominal circumference [AC], femur length [FL], nasal bone if GA 15–22 weeks and nuchal thickness if GA 16–20 weeks). Though not considered in defining whether an anatomy survey was complete or incomplete, we analyzed separately whether the 5 specified fetal measurements were recorded.

### 2.4. Statistical Analyses

Preliminary exploration was performed on data pooled from all practices combined. We plotted the percentage of inadequate views for each exam element as a function of GA for the entire dataset (GA range: 14 to 40 weeks). Subsequent analysis was restricted to GAs of 18.0 to 23.9 weeks for several reasons. First, several organizations recommend that the optimal time for anatomy surveys is between about 18–22 weeks GA for most patients [1,25,26,27,28]. Second, patients seen at 24 weeks GA or later are more likely to have been referred for known or suspected abnormalities rather than simply for screening. Third, 75% of all the exams were performed within this range of GAs, so the exclusion of earlier or later exams would not seriously degrade the sample size for the quality review. Finally, for most anatomy elements, the rate of inadequate views was lowest and fairly constant in this GA range.

For exams from 18.0 to 23.9 weeks GA, we tabulated the rate of normal, abnormal and inadequate views for each of the 36 required anatomy elements in all 7 practices combined. In the pooled data, we evaluated the associations between the rate of incomplete exams and maternal obesity (BMI ≥ 30 kg/m^2^), prior cesarean, advanced maternal age (≥ 35 years at the EDD) and GA < 19 weeks using the chi-squared test and univariable and multivariable logistic regression.

Next, we stratified the data by practice and tabulated the rate of incomplete exams, the rate of exams with 1 or more abnormal views, the number of inadequate views in the incomplete exams and the rates of missing fetal biometry measurements. Between-practice differences were assessed with the chi-squared test, Kruskal–Wallis test, and logistic regression, adjusting for maternal obesity, prior cesarean, maternal age and GA < 19 weeks.

Finally, we selected the practice with the highest rate of incomplete exams to demonstrate examples of individual-level comparisons between selected sonographers and between selected physicians.

Two-tailed *p*-values less than 0.05 were considered significant.

For practices that wish to adopt these methods using their own data, Supplemental Files 1–4 provide a spreadsheet file with sample data (.XLSX format), Stata “do-file” analysis scripts (Stata .DO format) and a text file of the scripts readable by those who do not have Stata (.DOCX format).

## 3. Results

### 3.1. Included Exams and Gestational Age Considerations

**Figure 1:**
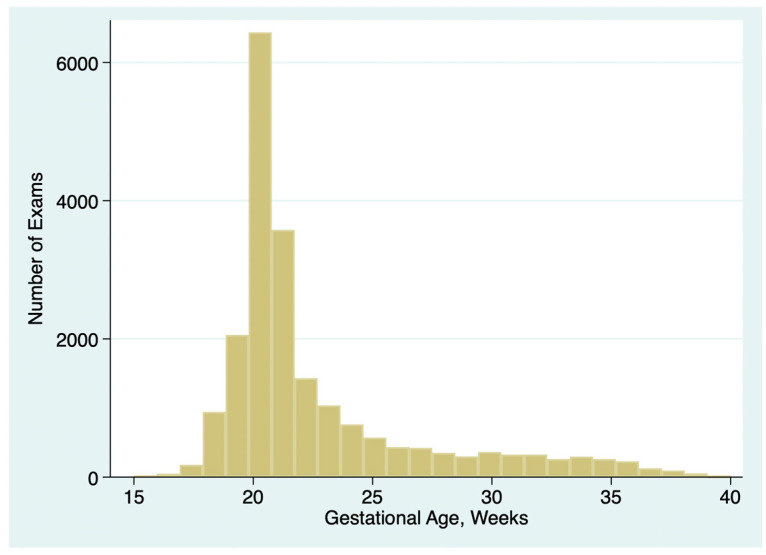
Number of detailed anatomy ultrasound exams performed in each gestational week; seven practices combined.

A total of 20,897 exams of 20,897 unique patients from the seven practices met the inclusion criteria. GA ranged from 14.0 to 39.7 weeks of gestation (median 21.0 weeks, interquartile range 20.0 to 23.6 weeks). The distribution of gestational ages is summarized in Figure 1.

**Figure 2:**
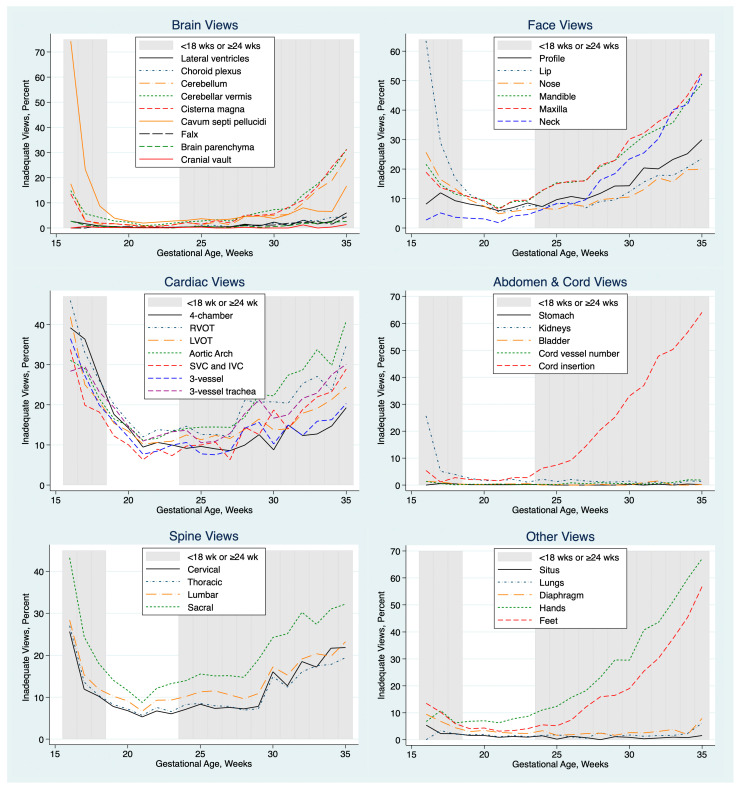
Percentage of exams with inadequate views of each structure. Unshaded area represents 18.0 to 23.9 weeks of gestation, range used for subsequent analyses. Data from 7 practices combined. IVC = inferior vena cava; LVOT = left ventricular outflow tract; RVOT = right ventricular outflow tract; SVC = superior vena cava, wks = weeks.

The percentage of exams with inadequate views of specific elements of the anatomy varied with GA, as shown in Figure 2. For most elements, the percentage was lowest and fairly constant between 18 and about 28 weeks GA. We focused our quality review on exams from 18.0 to 23.9 weeks of gestation for all subsequent analyses.

The percentages of normal, abnormal and inadequate views for each element of the anatomy exam at 18.0 to 23.9 weeks GA are shown in Table 1. The percentage of inadequate views varied by anatomical region, tending to be low for the fetal brain, lungs and abdomen and high for fetal heart, face and spine views.

### 3.2. Clinical Factors Associated with Incomplete Exams

**Figure 3:**
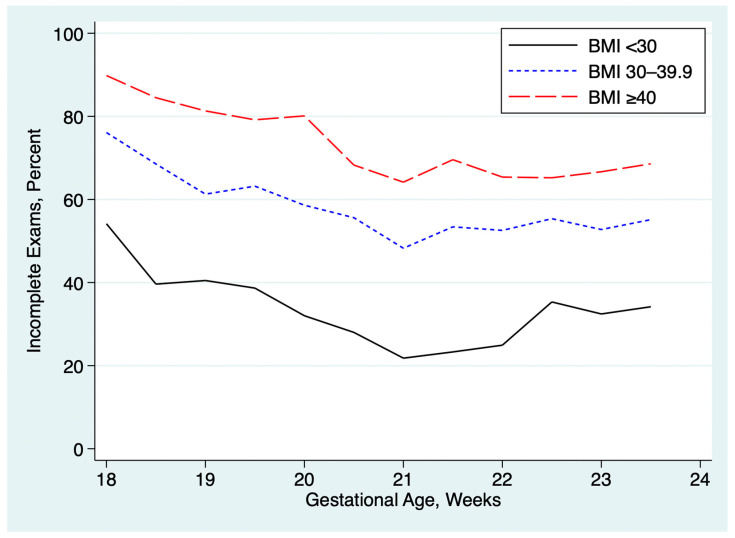
Associations between maternal obesity, gestational age and rate of incomplete exams. All practices combined. BMI = body mass index in kg/m^2^.

Table 2 summarizes the associations between incomplete exams and maternal obesity, prior cesarean delivery, advanced maternal age and gestational age at the time of the anatomy ultrasound exam. The rate of incomplete exams was higher in obese patients and rose with increasing BMI, as shown in Figure 3. Exams performed at 18.0 to 18.9 weeks GA were about twice as likely to be incomplete as exams performed at ≥19.0 weeks GA. Prior cesarean was associated with a small but statistically significant increased rate of incomplete exams. Advanced maternal age was associated with a slightly lower rate of incomplete exams, both in univariable analysis and after multivariable adjustment for the other factors.

### 3.3. Practice-Level Variation in Exam Completeness

The results stratified by practice are summarized in Table 3. There was wide variation between practices in the percentage of exams that were incomplete, ranging from 1% in one practice to 50% or more in two practices. There was also a wide variation in the percentage of exams in which an abnormality was identified, ranging from 2% to 10.0%.

**Figure 4:**
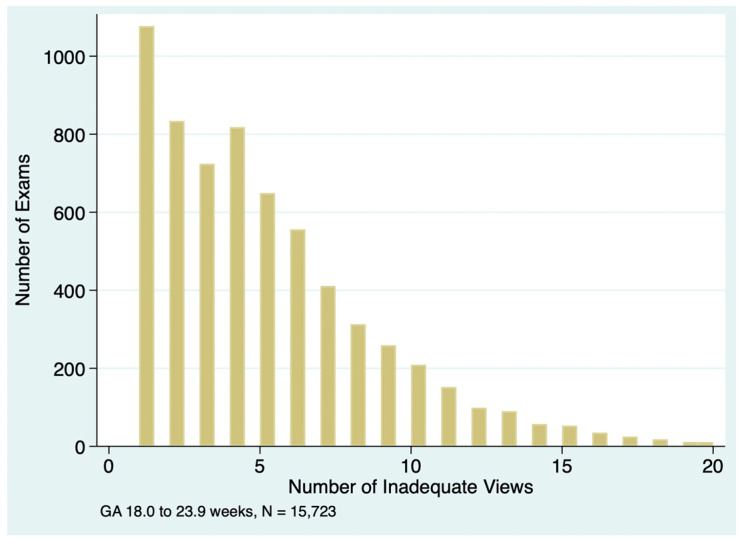
Number of inadequate views in exams with incomplete fetal anatomy.

If an anatomy exam was incomplete, there were often multiple inadequate views, as illustrated in Figure 4. The median number of inadequate views was four (IQR 2 to 7) in the incomplete exams across all practices combined, with significant variations between practices, as shown in Table 3. In exams with only one inadequate view, the most common elements coded as inadequate were the aortic arch (*n* = 135), venae cavae (*n* = 95), 3-vessel trachea view (*n* = 63) and profile (*n* = 41).

The basic fetal biometry measurements (BPD or HC, AC, FL) were recorded in virtually all the exams, with only four missing measurements in 20,897 exams (0.02%): three missing head measurements and one missing AC.

Nuchal fold measurement was not recorded in 2.8% of exams at ≤20 weeks GA, with significant variation between practices (ranging from 0 to 9.4% missing measurements). This measurement was missing in 20.1% of exams when the neck views were inadequate (35 of 174) versus 2.1% when neck views were adequate (109 of 4995, *p* < 0.001, Fisher’s exact text).

Nasal bone measurement was not recorded in 38.4% of exams at ≤22 weeks GA, again with significant variation between practices (ranging from 9.9% to 100% missing measurements). This measurement was missing in 85.3% of exams when the profile view was recorded as inadequate (840 of 985) versus 34.8% when the profile was adequate (4405 of 12,644, *p* < 0.001, Fisher’s exact test).

There was no evident relationship between the practice-level percentage of incomplete anatomy exams, missing nuchal fold measurements, or missing nasal bone measurements. For example, Practice 1 had the lowest rate of incomplete anatomy but the highest rate of missing nuchal fold and nasal bone measurements. Practice 7 had the second lowest rate of incomplete anatomy exams but one of the highest rates of missing nasal bone measurements and not a single case of a missing nuchal fold measurement.

### 3.4. Sonographer-Level Variation in Exam Completeness

Table 4 summarizes results from 10 of the 42 sonographers from a single practice. This practice and these sonographers were hand-selected to illustrate several types of quality issues that can be explored using our methods; they are not a random sample and are not intended to be comprehensive. Individual sonographers’ rates of incomplete exams varied from 13.6% to 83.2%. Each sonographer’s rate of incomplete exams was compared to the rate of the rest of the practice using logistic regression, adjusting for the factors in Table 3. In this analysis, Sonographers 1 through 3 had significantly lower rates of incomplete exams than the rest of the practice, and Sonographers 6 through 10 had significantly higher rates. There was also significant between-sonographer variation in the number of inadequate views in the incomplete exams, with medians ranging from 2 to 5 inadequate views per incomplete exam.

Measurements of nuchal fold thickness were missing in 0 to 16% of the exams, a significant between-sonographer variation. There was no evident relationship between a sonographer’s rate of incomplete anatomy exams and the rate of missing nuchal fold measurements. For example, Sonographers 8 and 10 had two of the highest rates of incomplete anatomy exams, but no missing nuchal fold measurements. In contrast, Sonographers 6 and 7 had intermediate rates of incomplete anatomy exams but the highest rates of missing nuchal fold measurement.

Measurements of nasal bone length were missing in 4.9 to 99.8% of exams, a significant between-sonographer variation. There was some tendency for sonographers with high rates of incomplete anatomy exams to also have high rates of missing nose bone measurement. For example, Sonographers 9 and 10 had the highest rate of incomplete anatomy exams and two of the highest rates of missing nasal bone measurements; Sonographer 1 had the lowest rate of both incomplete anatomy exams and missing nose bone measurement. Sonographer 5 was an exception to this general trend, with an intermediate rate of incomplete anatomy exams but a high rate of missing nose bone measurements. There was no obvious relationship between the rate of missing nuchal fold measurements and the rate of missing nasal bone measurements.

### 3.5. Physicain-Level Variation in Exam Completeness

Table 5 summarizes results from 7 of the 14 physicians from the same practice as the sonographers in Table 4. As with Table 4, these physicians were hand-picked to illustrate some of the quality issues that can be explored with our methods. Similarly to the results for the sonographers, there were significant between-physician differences in the rate of incomplete anatomy exams and the rates of missing measurements for the nuchal fold and nose bone. We evaluated the possibility that high physician workload may increase the rate of incomplete exams. As shown in Supplementary File S5, physicians with a higher mean daily volume of ultrasound procedures had lower rates of incomplete exams and exams with abnormalities.

### 3.6. Focused Review of Inadequate Elements by Selected Examiners

To explore which elements of the anatomy survey were often classified as inadequate, we focused on the two sonographers in Table 4 with the highest rates of incomplete exams. This practice has multiple offices across a large metropolitan area and the sonographers and physicians work in select offices preferentially. As a result, each sonographer tends to work with only a few physicians and vice versa. For example, 91% of the exams conducted by Sonographer 10 were signed by Physician 7, and 63% of the exams signed by Physician 7 were performed by Sonographer 10. Similarly, 90% of the exams conducted by Sonographer 9 were signed by Physician 2, and 40% of the exams signed by Physician 2 were performed by Sonographer 9. Thus, in Table 6, we include the rates of inadequate views for Sonographers 9 and 10 and for their most common reading physicians (Physicians 7 and 2, respectively).

The inspection of Table 6 reveals that both Sonographer 10 and Physician 7 had inadequate views of the fetal hands in >60% of exams, remarkably higher rates than Sonographer 9 and Physician 2 (both <5%). Sonographer 9 had inadequate views of the fetal maxilla, mandible and neck in >40% of exams, substantially higher rates than the other examiners shown.

## 4. Discussion

### 4.1. Principal Findings

The rate of incomplete anatomy exams and missing measurements varied widely between practices and between individual examiners within a practice.

Incomplete exams occur because of inadequate views of one or more specific elements of the anatomy. We found that views of fetal cardiac anatomy, face and spine were inadequate more often than views of the brain, abdominal organs and cord. This confirms prior reports suggesting that it is often more difficult to obtain adequate images of the heart, face and spine [5,6,12,15]. Our finding that incomplete exams were more common with maternal obesity, abdominal scarring associated with prior cesarean delivery and earlier gestational age also confirms prior reports [3,4,6,7,9,10,12,13,14,15,16].

### 4.2. Clinical Consequences of Incomplete Exams

If the anatomy is incompletely evaluated, it is possible that a congenital anomaly will be missed. For this reason, it is recommended that a repeat examination be performed to re-examine the anatomy views not adequately seen on the initial exam [16]. A single follow-up exam will usually yield adequate images of the views not seen on the index exam [5,7,9,12,14,15]. It is debatable whether repeated follow-up exams improve completion rates if the anatomy evaluation remains incomplete after the first follow-up [5,8,9,12,15,29,30]. This is especially problematic with maternal obesity: 11% of patients with a BMI > 30 kg/m^2^ and 25% of patients with a BMI > 50 kg/m^2^ failed to have a complete evaluation of fetal anatomy by the time of birth, despite multiple repeated attempts [16,29].

One study found that inadequate views were associated with an increased incidence of congenital anomalies [12], but this was not confirmed by others [7,15]. If an anomaly is found, management options depend on the GA at which the diagnosis is suspected or confirmed. Detection at the earliest possible GA usually provides the most options for confirmatory testing, advanced imaging, such as fetal echocardiogram or magnetic resonance imaging, and pregnancy termination.

Even if the anatomy survey is ultimately completed and found to be normal, follow-up exams are inevitably costly to the patient in terms of time and inconvenience and potentially have direct financial costs for patients or payers. Incomplete anatomy exams also cause significant patient anxiety [11].

### 4.3. Ideal Rate of Incomplete Exams

There is no benchmark or “gold standard” to establish an ideal rate of incomplete exams. Rates reported in prior studies vary widely, from less than 4% [5] to 57% [4], with several studies reporting rates in the range of 10–20% [7,8,9,11,14].

When using ultrasound to screen for congenital anomalies, there is an inevitable trade-off between sensitivity and specificity. To maximize sensitivity for the detection of anomalies, sonographers or physicians may be unwilling to call a view adequate unless it is very clear. The higher an examiner’s threshold for deciding that a view is adequate, the more likely that examiner is to finish the exam with inadequate views, rendering the exam incomplete and resulting in the recommendation of a follow-up exam. While this will increase sensitivity, it decreases specificity; that is, incomplete exams become non-specific findings, resulting in the need for a follow-up exam for many patients with normal fetuses.

Unexplained variation between examiners or between practices can lead to bias and may be an indicator of a quality problem [31]. Outlier providers or outlier practices, by definition, are those that deviate from the “norm” established by providers or practices whose rate of incomplete exams falls closer to the middle. Closer scrutiny of the adequacy of images may be warranted for these outliers. For example, the 1.3% rate of incomplete exams in Practice 1 may reflect a predisposition to accept as “normal” some images that other examiners might find suboptimal; the low rate of reported abnormal views in this practice reinforces that notion. For Practice 2, on the other hand, the 52.7% rate of incomplete exams may reflect a practice-wide tendency to call views “suboptimal” even though other examiners may accept those views as adequate for diagnostic purposes. In the absence of a benchmark, it is not possible to conclude that one approach is better than the other.

On the other hand, there is clearly a quality problem if a high percentage of measurements of the nuchal fold or nasal bone are missing. Increased nuchal thickness is one of the strongest sonographic markers of Down syndrome [32,33] and is also associated with other chromosomal anomalies [34,35,36,37]. A short or absent nose bone is associated with Down syndrome, other aneuploidies and a variety of other anomalies [32,38,39]. We believe these measurements should be obtainable in virtually all exams at ≤22 weeks GA, with occasional exceptions due to fetal position or extremely high maternal BMI.

### 4.4. Possible Strategies to Reduce the Rate of Incomplete Exams

One strategy to reduce incomplete exams might be to avoid scheduling detailed anatomy exams before 19 weeks of gestation. Although several organizations recommend that the routine anatomy exam be performed at 18–22 weeks [25,26,27,28], some fetal anatomy elements are difficult to image adequately during week 18, and we found that exams at GAs of 18.0 to 18.9 weeks were incomplete about twice as often as exams at GAs of 19.0 to 23.9 weeks.

However, improving the completion rate by postponing exams must be balanced against the desire to detect congenital anomalies as early as possible in gestation in order to allow sufficient time for counseling and genetic evaluation before the age of fetal viability. Despite a high rate of incomplete exams at early GAs, detailed anatomy evaluation can detect 37% of structural anomalies and 91% of lethal anomalies at GAs of 11 to 14 weeks [40] and 59% of structural anomalies at GAs of 14–17 weeks [41]. Perhaps the strategy that will best optimize the early detection of anomalies while minimizing incomplete evaluations would be to perform two detailed anatomy evaluations, one in the first trimester and one in the mid-second trimester, though the optimal timing of the two exams in a two-step strategy is not currently known.

Another strategy to reduce the rate of incomplete exams might be to increase the amount of time routinely allotted for a detailed anatomy exam. There is no established benchmark or standard regarding the time that should be allotted, so practices generally set their schedules based on historic precedent. But the number of required views has increased over the past decades, so perhaps the allotted time should be proportionately increased. One study found that increasing the allotted time from 30 minutes to 45 minutes was associated with a decrease in the rate of incomplete exams from 46% to 20% and a significant reduction in the rate of repeat exams to complete anatomy surveys [17]. Increasing the scheduled exam time to 60 minutes might reduce the rate even further. For example, Practice 7, which had one of the lowest rates of incomplete exams in our cohort, allots 60 minutes for a routine detailed exam and 70 minutes if a transvaginal cervical length assessment is anticipated because of a prior spontaneous preterm birth.

Another strategy to reduce the rate of incomplete exams might be for physicians to “backscan” exams with inadequate views, that is, to repeat some of the exam after the sonographer has finished. Physicians might be able to obtain views that sonographers could not, either by virtue of having more experience or because the fetus may have moved to a more favorable position. Some of our physicians routinely “backscan” certain views or routinely attempt to clear inadequate views. Unfortunately, there is no information in the Viewpoint database to indicate which images were obtained by sonographers and which were obtained by physicians, so we do not know how often such “backscanning” was performed.

### 4.5. Evaluation of Individual-Level Variance

A prior study found that sonographer-level and physician-level variation was responsible for much of the variance in the rate of incomplete anatomy evaluation and the rate of recommendation for repeat examination [15]. We confirm that there is a large variance between examiners in the rate of incomplete exams.

Without performing a quantitative quality review, sonographers and physicians have no way of knowing how their performance compares to their peers. The practice-wide assessment of exam completion, as illustrated in Table 4 and Table 5, provides a high-level overview of individual performance. As with many quality review processes, the initial reaction of many providers is often denial that there is an issue; providers often believe that their patients are at a higher risk for incomplete exams for one reason or another. For that reason, we performed a multivariable adjustment for the known covariates: obesity, prior cesarean, maternal age and GA < 19 weeks. Even after adjustment, statistically significant differences between examiners persisted.

As shown by the examples in Table 6, a detailed individual assessment of inadequate views may yield insights into elements of the anatomy exam where examiners are uncomfortable and may need additional training or supervision. For example, Sonographer 9 appears to have an issue with views of the maxilla, mandible and neck.

The high rate of inadequate views of the hands for Sonographer 10 and Physician 7 is more difficult to interpret. It may be that Sonographer 10 is having trouble imaging the hands or it may be that Physician 7 has a low threshold to call hand images inadequate. A clue that the high rate of inadequate hand images was being driven by the physician is that over one-third of Physician 7′s exams were performed by other sonographers, yet the Physician’s overall rate was still >60%, meaning that this physician’s exams had a high rate of inadequate hand views no matter which sonographer performed the exam. An image review may yield further insights.

We suggest that sonographers and physicians should be rotated so that each sonographer has the opportunity to work with multiple physicians and vice versa. Working with different people may help to minimize the development of bad habits.

### 4.6. Strengths and Limitations

A strength of our quantitative approach is that it provides a broad overview of the performance of an entire practice and of individual personnel. For examiners with a large number of exams, the method is highly sensitive to small between-provider variations, readily identifying outliers for focused review. Once the analysis script is written, the quantitative analysis can be performed rapidly and repeated periodically. For those who wish to use these methods, we have provided a sample spreadsheet with sample data (Supplementary File S1) and sample Stata scripts (Supplementary Files S2 and S3) that will perform the basic analyses shown in Table 1, Table 2, Table 3, Table 4, Table 5 and Table 6 and generate graphs like Figure 1, Figure 2, Figure 3 and Figure 4.

A limitation is that the analysis is based entirely on findings stated in the reports rather than a review of the adequacy of the images. An audit of images is recommended to evaluate outlier personnel [19], but we believe that a labor-intensive, time-consuming image audit is unlikely to be informative for the majority of examiners whose performance is in line with others in their practice. Further, we believe that a report showing the exam as incomplete likely reflects a truly incomplete exam. Because Viewpoint offers an option to select “all normal” for the anatomy elements, a complete exam is typically the default condition. It requires a deliberate action to convert an element to anything other than normal. This is unlikely to happen unless the sonographer or physician truly finds the view to be abnormal or suboptimal.

Ultimately, the true accuracy of the fetal anatomy exam should be measured by the rate of correct determination of the presence or absence of congenital anomalies at birth or in infancy. But there are serious practical limitations to evaluating accuracy this way. The first limitation is a sample size consideration: Let us assume that the incidence of anomalies is about 4%, and one examiner has a detection rate of 75% of anomalies (or 3% of exams) while another has a detection rate of 25% of anomalies (or 1% of exams), a 3-fold difference in detection rate. To have 80% power for this difference to reach statistical significance at *p* < 0.05, a sample of 769 exams per sonographer would be required. This is more than double the number of exams performed annually by our busiest sonographers. The second limitation is that accuracy assessment for anomaly detection would require considerable effort to obtain newborn data from the several hospitals throughout a wide region where our referring providers deliver their patients. The third limitation is that some anomalies might be diagnosed later in infancy and may not be evident in the newborn hospital record.

### 4.7. Future Directions—Software Enhancements

Viewpoint and other ultrasound reporting software can be configured to require mandatory responses in many exam fields. It would be useful if nuchal fold and nasal bone measurements could be so configured, thereby greatly reducing the rate of exams missing these measurements. These measurements are only required at a restricted range of GAs (16–20 weeks for nuchal fold, 15–22 weeks for nasal bone). Unfortunately, Viewpoint cannot currently be configured to make an entry mandatory only in certain GA ranges. Future versions of the software should allow this, compelling users to either enter a measurement or to indicate that a valid measurement could not be obtained because of fetal position or another issue.

Reporting software should include fields to record all the required elements of the exam with simple choices such as normal, abnormal, suboptimal, not seen, etc. We found that Viewpoint did not have such fields for specific elements (position and architecture of legs and arms, shape and curvature of spine, integrity of spine and overlying tissue). Although we are confident that physicians would comment on abnormalities of each of these if present, we recommend that Viewpoint add these to the list of standard views in the anatomy software data-entry templates.

It would be ideal if software developers would include quality review tools in future versions of their product. Straightforward tools to evaluate practice-wide and provider-specific rates of incomplete exams and inadequate views would be useful. Although our sample data and Stata scripts in Supplementary Files S1–S4 may save practices the considerable time it would take to develop and debug analysis routines, there is still time and effort involved in querying the database, downloading the results, exporting them to spreadsheets and importing them into Stata. Other quality review tools might also be built into the reporting software, such as a provider-specific quantitative review of fetal biometry measurements and the accuracy of estimated fetal weight, as we outlined in prior reports [22,23].

Finally, software developers are currently directing considerable effort to developing artificial intelligence (AI) techniques to guide sonographers through exams and to help physicians determine whether specific views are normal, abnormal, or inadequate. We hypothesize that use of AI will decrease the rate of incomplete exams. This hypothesis needs to be tested as new suites of AI software are introduced.

## 5. Conclusions

There is considerable variation between practices and between examiners in the rate of incomplete anatomy exams and the rate of missing nuchal fold and nasal bone measurements. For examiners with a high rate of incomplete exams, tabulation of which views are inadequate may help to guide education and quality improvement. Some of our practices and some individuals need a quality improvement effort starting with feedback and education regarding the importance of measuring the nuchal fold and nose bone as a standard part of the second-trimester anatomy exam.

We believe it is important for every ultrasound practice to perform a quality review such as this. We have demonstrated a method and provided tools to allow practices to perform a similar evaluation.

## Figures and Tables

**Table 1:** Reported findings for each fetal structure at 18.0 to 23.9 weeks of gestation.

Structure	AIUM Item Number	Normal %	Abnormal *n* (%)	Inadequate %
Situs	6	98.6%	6 (0.04%)	1.4%
Lateral cerebral ventricles	8	99.2%	71 (0.45%)	0.3%
Choroid plexus	9	99.2%	91 (0.58%)	0.3%
Midline falx	10	99.5%	12 (0.08%)	0.45%
Brain parenchyma	11	99.7%	14 (0.09%)	0.2%
Cerebellum	15	98.5%	39 (0.25%)	1.2%
Cerebellar vermis including 4th ventricle	16	97.8%	22 (0.14%)	2.1%
Cisterna magna	17	98.7%	19 (0.12%)	1.2%
Cavum septi pellucidi	18	96.7%	25 (0.16%)	3.1%
Cranial vault, integrity and shape	19	99.7%	26 (0.17%)	0.1%
Profile (mid-sagittal)	20	92.3%	55 (0.35%)	7.3%
Upper lip (coronal view)	21	91.0%	27 (0.17%)	8.8%
Nose (coronal view)	22	92.4%	38 (0.24%)	7.4%
Maxilla (axial view)	24	90.8%	8 (0.05%)	9.1%
Mandible (axial view)	25	90.8%	3 (0.02%)	9.1%
Neck	26	96.8%	3 (0.02%)	3.1%
Lungs	28	98.2%	16 (0.10%)	1.7%
Diaphragm	29	96.8%	13 (0.08%)	3.1%
Hands	31	92.9%	21 (0.13%)	7.0%
Feet	32	95.6%	57 (0.36%)	4.1%
Stomach	33	99.7%	25 (0.16%)	0.2%
Kidneys	34	95.5%	401 (2.6%)	1.8%
Urinary bladder	35	99.6%	8 (0.05%)	0.3%
Umbilical cord, number of vessels	36	98.7%	159 (1.01%)	0.3%
Umbilical cord, insertion into abdomen	37	97.8%	12 (0.08%)	2.1%
4-chamber view of heart	39	84.8%	177 (1.13%)	14.1%
Right outflow tract	40	83.7%	34 (0.22%)	16.1%
Left outflow tract	41	86.1%	64 (0.41%)	13.2%
Aortic arch (sagittal view)	42	85.4%	32 (0.20%)	14.4%
Superior and inferior vena cavae	43	90.0%	19 (0.12%)	9.9%
3-vessel view	44	87.8%	57 (0.36%)	11.8%
3-vessel trachea view	45	84.8%	56 (0.36%)	14.9%
Cervical spine	47	93.1%	5 (0.03%)	6.8%
Thoracic spine	48	92.5%	8 (0.05%)	7.3%
Lumbar spine	49	90.8%	18 (0.11%)	9.0%
Sacral spine	50	87.9%	16 (0.10%)	12.0%

Examinations from all 7 practices combined, total *n* = 15,723. AIUM Item Number refers to American Institute of Ultrasound in Medicine case study submission requirements for accreditation in detailed 2nd trimester OB examination [2].

**Table 2:** Association of incomplete exams with several clinical factors.

Characteristic	Incomplete Exams, *n*/*N* (%)	Univariable Odds Ratio (95% Confidence Interval)	Multivariable Odds Ratio (95% Confidence Interval)
Maternal obesity **§**			
BMI < 30 kg/m^2^	3424/10,918 (31.2%)	Referent	--
BMI ≥ 30 kg/m^2^	2992/4805 (62.3%)	3.61 (3.36–3.88)	3.56 (3.32–3.83)
Gestational age **§**			
18.0–18.9 weeks	640/1122 (57.0%)	2.03 (1.79–2.29)	2.11 (1.86–2.40)
19.0–23.9 weeks	5776/14,601 (39.6%)	Referent	--
Prior cesarean **§**			
Yes	1231/2616 (47.1%)	1.36 (1.25–1.48)	1.22 (1.11–1.33)
No	5285/13,107 (39.6%)	Referent	--
Maternal age **§**			
<35 years	4443/10,561 (42.2%)	Referent	--
≥35 years	1973/5182 (38.1%)	0.84 (0.79–0.90)	0.89 (0.83–0.95)

Seven practices combined, 18.0 to 23.9 weeks of gestation. Multivariable model was logistic regression including all factors shown. **§—***p* < 0.001, significant difference between groups, Fisher’s exact test.

**Table 3:** Findings in detailed anatomy exams at 7 practices.

	Practice 1	Practice 2	Practice 3	Practice 4	Practice 5	Practice 6	Practice 7	Total
**Exams at GA 18.0 to 23.9 weeks**								
Number of anatomy exams	235	8303	1396	513	724	1534	3018	15,723
Incomplete exams, %	1.3% **†**	52.7% **†**	37.0% **†**	37.2% **†**	30.5% **†**	49.7% **†**	11.4% **†**	41%
Number of inadequate views								
in incomplete exams,	2 (1–5)	4 (2–7)	3 (2–5)	4 (2–6)	3 (1–5)	5 (2–8)	4 (2–6)	4 (2–7)
median (interquartile range) **§**								
Exams with ≥1 abnormal	1.7%	7.2%	10.0%	9.8%	5.5%	7.0%	5.2%	7.0%
anatomy view, % **‡**								
**Exams at GA ≤ 20.0 weeks**								
Number of exams	64	2969	493	244	433	698	268	5169
Nuchal fold not measured, % **‡**	9.4%	3.3%	5.7%	1.6%	0.7%	0.6%	0	2.8%
**Exams at GA ≤ 22.0 weeks**								
Number of exams	204	7023	1077	406	646	1433	2860	13,649
Nasal bone not measured, % ‡	100%	19.2%	40.7%	9.9%	91.5%	56.1%	63.7%	38.4%

**†**—*p* <0.001; significantly different than other practices combined; logistic regression adjusting for maternal obesity, prior cesarean, maternal age and GA < 19 weeks. **‡**—*p* < 0.001, significant difference between practices, chi-squared test. **§—***p* < 0.001, significant difference between practices, Kruskal–Wallis test. Abbreviations: AC = abdominal circumference; BPD = biparietal diameter; BMI = body mass index; FL = femur length; GA = gestational age; HC = head circumference; IQR = interquartile range; SD = standard deviation.

**Table 4:** A comparison of selected sonographers from a single practice.

Sonographer	Incomplete Anatomy Exams, *n*/*N* (%)	Number of Inadequate Views If Anatomy Incomplete, Median (IQR) †	Nuchal Fold Not Measured at 18.0–20.0 Weeks, *n*/*N* (%)	Nasal Bone Not Measured at 18.0–22.0 Weeks, *n*/*N* (%)
Sonographer 1	44/317 (13.8%) **‡**	2 (1–5)	0/92 (0%)	13/265 (4.9%) **§**
Sonographer 2	70/348 (20.1%) **‡**	2 (1–4)	1/105 (1%)	16/284 (5.6%) **§**
Sonographer 3	106/256 (41.4.6%) **‡**	3 (2–5)	2/70 (3%)	44/223 (19.7%)
Sonographer 4	127/269 (47.2%)	5 (3–7)	2/84 (2%)	20/219 (9.1%) **§**
Sonographer 5	76/156 (48.7%)	2 (1–6)	3/58 (5%)	76/132 (57.6%) **§**
Sonographer 6	184/300 (61.3%) **‡**	5 (3–7)	11/113 (10%) **§**	48/257 (28.7%)
Sonographer 7	104/157 (66.2%) **‡**	4 (2–7)	9/56 (16%) **§**	32/133 (24.1%)
Sonographer 8	209/275 (76.0%) **‡**	5 (3–7)	0/104 (0%) **§**	28/246 (11.4%) **§**
Sonographer 9	248/310 (80.0%) **‡**	6 (3–8)	3/97 (3%)	259/261 (99.2%) **§**
Sonographer 10	267/321 (83.2%) **‡**	4 (2–7)	0/83 (0%)	136/264 (51.3%) **§**
Practice Total	4379/8303 (52.7%)	4 (2–7)	99/2969 (3.3%)	1346/7023 (19.2%)

Exams at 18.0 to 23.9 weeks of gestation. Practice totals include 32 additional sonographers not shown. **‡**—*p* < 0.05; significantly different than remainder of practice; logistic regression adjusted for maternal obesity, prior cesarean, maternal age and GA <19 weeks. **†**—*p* <0.001, significant difference between sonographers, Kruskal–Wallis test. **§—***p* < 0.05, significantly different than remainder of practice, Fisher’s exact test.

**Table 5:** A comparison of selected physicians from the same practice as Table 4.

Physicians	Incomplete Exams, *n*/*N* (%)	Number of Inadequate Views If Anatomy Incomplete, Median (IQR) †	Nuchal Fold Not Measured At 18.0–20.0 Weeks, *n*/*N* (%) ‡	Nasal Bone Not Measured At 18.0–22.0 Weeks, *n*/*N* (%) ‡
Physician 1	248/678 (36.6%) **§**	4 (2–5)	8/206 (3.9%)	78/572 (13.6%) **‡**
Physician 2	416/983 (42.3%) **§**	5 (2–8)	9/322 (2.8%)	239/803 (29.4%) **‡**
Physician 3	298/604 (48.0%) **§**	4 (2–5)	1/193 (0.5%) **‡**	73/529 (14.1%) **‡**
Physician 4	361/711 (50.8%)	5 (3–8)	8/272 (2.9%)	92/591 (15.6%) **‡**
Physician 5	162/278 (58.3%) **§**	5 (3–8)	0/75 (0%)	30/238 (12.6%) **‡**
Physician 6	413/567 (72.8%) **§**	6 (3–9)	5/172 (2.9%)	82/501 (16.4%)
Physician 7	382/511 (74.8%) **§**	3 (1–5)	4/154 (2.6%)	211/426 (49.5%) **‡**
Practice Total	4359/8303 (52.5%)	4 (2–7)	99/2969 (3.3%)	1345/7023 (19.2%)

Exams at 18.0 to 23.9 weeks of gestation. Practice totals include 7 additional physicians not shown. **‡**—*p* < 0.05; significantly different than remainder of practice; logistic regression adjusted for maternal obesity, prior cesarean, maternal age and GA <19 weeks. **†**—*p* <0.001, significant difference between physicians, Kruskal–Wallis test. **§—***p* < 0.05, significantly different than remainder of practice, Fisher’s exact test.

**Table 6:** Focused review: Rate of inadequate views by selected examiners, exams at 18.0 to 23.9 weeks of gestation.

Structure	Sonographer 10 (321 Exams) Inadequate Views, *n* (%)	Physician 7 (511 Exams) Inadequate Views, *n* (%)	Sonographer 9 (310 Exams) Inadequate Views, *n* (%)	Physician 2, (983 Exams) Inadequate Views, *n* (%)
Situs	3 (0.9%)	1 (0.2%)	7 (2.3%)	18 (1.6%)
Lateral cerebral ventricles	1 (0.3%)	2 (0.4%)	1 (0.3%)	10 (1.0%)
Choroid plexus	1 (0.3%)	2 (0.4%)	4 (1.3%)	6 (0.6%)
Midline falx	0	1 (0.2%)	0	8 (0.8%)
Brain parenchyma	8 (2.4%)	10 (2.0%)	0	0
Cerebellum	8 (2.4%)	10 (2.0%)	1 (0.3%)	10 (1.0%)
Cerebellar vermis including 4th ventricle	12 (3.7%)	17 (3.3%)	1 (0.3%)	14 (1.4%)
Cisterna magna	4 (1.3%)	6 (1.2%)	0	10 (1.0%)
Cavum septi pellucidi	24 (7.5%)	28 (5.5%)	4 (1.3%)	25 (2.5%)
Cranial vault, integrity and shape	0	0	0	2 (2.0%)
Profile (mid-sagittal)	53 (16.5%)	67 (13.1%)	27 (8.7%)	59 (6.0%)
Upper lip (coronal view)	50 (15.6%)	59 (11.6%)	50 (16.1%)	87 (8.9%)
Nose (coronal view)	41 (12.8%)	54 (10.6%)	40 (12.9%)	77 (7.8%)
Maxilla (axial view)	82 (25.6%)	118 (23.9%)	148 (47.7%)	157 (16.0%)
Mandible (axial view)	77 (24.0%)	115 (22.5%)	150 (48.4%)	169 (17.2%)
Neck	23 (7.2%)	23 (4.5%)	127 (41.0%)	102 (10.4%)
Lungs	0	1 (0.2%)	68 (21%)	65 (6.6%)
Diaphragm	7 (2.2%)	6 (1.2%)	12 (3.9%)	41 (4.1%)
Hands	213 (66.4%)	320 (62.6%)	15 (4.8%)	21 (2.1%)
Feet	35 (10.9%)	42 (8.2%)	9 (2.9%)	24 (2.4%)
Stomach	0	0	1 (0.3%)	1 (0.1%)
Kidneys	8 (2.5%)	11 (2.2%)	11 (3.6%)	20 (2.0%)
Urinary bladder	1 (0.3%)	0	3 (1.0%)	4 (0.4%)
Umbilical cord, number of vessels	0	0	0	1 (0.1%)
Umbilical cord, insertion into abdomen	7 (2.1%)	8 (1.6%)	8 (2.6%)	14 (1.4%)
4-chamber view of heart	95 (29.6%)	110 (23.1%)	41 (13.2%)	108 (11.0%)
Right outflow tract	25 (7.8%)	21 (4.1%)	91 (29.4%)	146 (14.9%)
Left outflow tract	28 (8.7%)	26 (5.1%)	69 (22.3%)	135 (13.7%)
Aortic arch (sagittal view)	100 (31.2%)	123 (24.1%)	108 (34.8%)	160 (16.2%)
Superior and inferior vena cavae	22 (6.9%)	6 (1.2%)	49 (15.8%)	81 (8.2%)
3-vessel view	18 (5.6%)	30 (5.9%)	56 (18.1%)	125 (12.7%)
3-vessel trachea view	51 (15.9%)	69 (13.5%)	86 (27.4%)	152 (15.5%)
Cervical spine	20 (6.2%)	23 (4.5%)	71 (22.9%)	100 (10.2%)
Thoracic spine	11 (3.4%)	12 (2.4%)	71 (22.9%)	100 (10.2%)
Lumbar spine	25 (7.8%)	30 (5.9%)	76 (24.5%)	111 (11.3%)
Sacral spine	57 (17.8%)	67 (13.1%)	87 (28.1%)	126 (12.8%)

## Data Availability

The raw datasets presented in this article are not readily available because they include protected patient health information and the names of individual healthcare personnel. As an alternative, we provide a sample dataset in Supplementary File S1 with anonymized patient and personnel identifiers and random jitter added to dates and measurements. The purpose of providing these data is to facilitate the development and debugging of data analysis programs like those provided in Supplementary Files S2 and S3. Requests to access the original datasets should be directed to the corresponding author.

## Supplementary Materials

The following supporting information can be downloaded at: https://www.mdpi.com/article/10.3390/jcm14103356/s1. Supplementary File 1: “EXCEL Anatomy & Indications.xlsx”; Supplementary File 2: “DO-FILE Anatomy Quality Review.do”; Supplementary File 3: “DO-FILE-Itemrecode.do”; Supplementary File 4: “WORD version of Stata scripts.docx”; Supplementary File 5. “Physician Workload.pdf”.

## Abbreviations

The following abbreviations are used in this manuscript:

AC	Abdominal circumference (fetal)
AIUM	American Institute of Ultrasound in Medicine
BMI	Body mass index
BPD	Biparietal diameter (fetal)
DOB	Date of birth (maternal)
EDD	Estimated date of delivery (date when gestational age = 40 weeks)
FL	Femur length (fetal)
GA	Gestational age
HC	Head circumference (fetal)
IQR	Interquartile range
SD	Standard deviation
