# Supplementary material for: Quantitative Approach to Quality Review of Prenatal Ultrasound Examinations: Incomplete Detailed Fetal Anatomy Exams
Source: J Clin Med. 2025 May 12;14(10):3356. doi: 10.3390/jcm14103356 (PMC12112216; doi:10.3390/jcm14103356)
Supplement: Supplementary file 1 [file jcm-14-03356-s001.zip › Supplementary files/Supplementary File 4 - WORD version of Stata scripts.docx]

**Word Versions of Stata do-file scripts**

Supplement to Quantitative Approach to Quality Review of Prenatal Ultrasound Examinations: Incomplete Fetal Anatomy Survey. Combs et al. for Journal of Clinical Medicine 2025.

This Word .DOCX file shows the Stata scripts for the included Stata .DO files.

The .DO files can only be read using Stata software.

The Word file will not run in Stata.

It is provided so that readers who do not own Stata can see how the analysis was done.

You can use the provided .DO files to perform a quality review on your own data if you structure your Excel data file with a sheet named Anatomy and a sheet named Indications and with column headers and file name the same as the provided sample data file: “EXCEL Anatomy and Indications.xlsx”

**Contents**

Pages 2 through 21: Script for the main do-file, “DO-FILE Anatomy Quality Review.do”

Page 22: Script for the subroutine, “DO-FILE-itemrecode.do” which is called multiple times from the main do-file.

**DO-FILE Anatomy Quality Review.do**

**** Supplemental file for the Quantitative Approach to Quality Review of Prenatal Ultrasound Examinations: Incomplete Fetal Anatomy"

* Lines that start with Asterisk (*) are comments, not executed.

* Lines that start without Asterisk are Stata commands

* Lines that end with /// indicate that the command is continued on the following line.

***** INSTRUCTIONS *****

* A. You will need to have Stata version 13 or later installed on your computer

* B. Copy THREE files to the same folder on your computer

* 1. The Stata do-file - "DO-FILE Anatomy Quality Review.do" (this file)

* 2. The Stata do-file - "DO-FILE-itemrecode.do"

* 3. The Excel file - "EXCEL Sample Data - Anatomy and Indications.xlsx"

* C. Customize this file in a few places

* Line 23 - change the instruction to match the directory structure of your compute

* Lines 44++ - change to match how Obesity is recorded in your data

* Lines 65++ - change to match how Prior Cesarean is recorded in your data

* Lines 497++ - change to names of sonographers you want to tabulate

* Lines 549++ - change to names of physicians you want to tabulate

* Line 583 - change to name of the examiner you want to focus on

* Line 623 - change to name of the examiner you want to focus on

* D. Save your revisions (Save icon above) then click Do icon at upper right.

* The "cd" command below "cd" means Change Directory, making this the default directory.

* You will need to CUSTOMIZE the directory name to match the directory structure of your computer.

cd "/Users/andrewcombs/Desktop/Anatomy QR/"

log using "LOG Anatomy Quality Review", replace

* The Stata Log file is where the results of the analysis will appear.

version 13

set more off

**** INDICATIONS: OBESITY

*This section extracts Obesity info from the Indications file.

import excel "EXCEL Anatomy & Indications.xlsx", sheet("Indications") firstrow clear

*The next line replaces Indication with all lower-case characters

replace Indication = lower(Indication)

save "DATA Indications.dta", replace

* The next command tabulate s all indications that contain the string "obes"

* The strpos() function returns the position within Indication where the string occurs.

* If the string does not occur, strpos() will be 0.

* So strpos()>0 means only that the string is preseent.

tabulate Indication if strpos(Indication,"obes")>0

* The next lines create a new variable Obese, ands fill it in depedending on values of Indication

* You may need to CUSTOMIZE these lines depending on how Obesity is entered in Indications in your practice

generate Obese=""

replace Obese="BMI 30-39.9" if strpos(Indication,"bmi 3")>0

replace Obese="BMI 30-39.9" if strpos(Indication, "35")>0

replace Obese="BMI 40+" if strpos(Indication,"40")>0

* The next line deletes records that do not list Obesity

drop if Obese==""

* The next lines keep only the first entry for exams with more than one entry and saves a temporary data file with results

bysort ExamID: generate nExam=_n

keep if nExam==1

save "TEMP DATA Obesity.dta", replace

**** INDICATIONS: PRIOR CESAREAN

*This section extracts Prior Cesarean info from the Indications file.

use "DATA Indications.dta", clear

* The next command tabulate s all indications that contain the string "previous c-section"

* The strpos() function returns the position within Indication where the string occurs.

* If the string does not occur, strpos() will be 0.

* So strpos()>0 means only that the string is preseent.

tabulate Indication if strpos(Indication,"previous c-s")>0

* The next lines create a new variable PriorCS, ands fill it in depedending on values of Indication

* You may need to CUSTOMIZE these lines depending on how Prior Cesarean is entered in Indications in your practice

generate PriorCS=0

replace PriorCS=1 if strpos(Indication,"previous c-section")>0

* The next line deletes records that do not list Obesity

drop if PriorCS==0

* The next lines keep only the first entry for exams with more than one entry and saves a temporary data file with results

bysort ExamID: generate nExam=_n

keep if nExam==1

save "TEMP DATA Prior CS.dta", replace

***** ANATOMY DATA

import excel "EXCEL Anatomy & Indications.xlsx", sheet("Anatomy") firstrow clear

*** This section merges the indications Obese and PriorCS

merge 1:1 ExamID using "TEMP DATA Obesity.dta"

keep if _merge==1 | _merge==3

drop _merge

replace Obese="BMI <30" if Obese==""

merge 1:1 ExamID using "TEMP DATA Prior CS.dta"

keep if _merge==1 | _merge==3

drop _merge

replace PriorCS=0 if PriorCS==.

*The following lines check that only Detailed exams, finalized exams, and exams with cardiac activity are included.

tabulate Examstatus

drop if strpos(Examstatus,"Scan started")>0

tabulate Examtype

keep if strpos(Examtype,"Detailed")>0

tabulate Cardiacactivity

keep if trim(Cardiacactivity)=="present"

drop Examstatus Examtype Cardiacactivity

* This section looks for patients with more than 1 exam and keeps only the first exam.

* After doing this, the PersonNumber identifier is deleted

bysort PersonNumber: generate nPerson=_n

tabulate nPerson

keep if nPerson==1

drop PersonNumber

*This section calculates maternal age at the EDD, gestational age at time of exam)

*After doing this, Date of Birth and EDD are deleted.

generate MatAge=(AssignedEDD-DOB)/365.25

generate GA = 40 - (AssignedEDD - Examdate)/7

drop DOB AssignedEDD

* This section renames some of the variables to reduce typing.

* Viewpoint has some field names that start with a number (4-chamberview, 3-vessel view, 3-vesseltracheaview)

* Stata automatically drops the leading number from the variable name, so below we add "Four" and "Three" to these variables.

rename Readingphysician Doc

rename Sonographer Tech

rename Lateralventricles LatVents

rename Midlinefalx Falx

rename Choroidplexus Choroid

rename Cisternamagna Cisterna

rename Cavumseptipellucidi Cavum

rename chamberview FourChamber

rename LVOTview LVOT

rename RVOTview RVOT

rename Aorticarchview AoArch

rename vesselview ThreeVV

rename vesseltracheaview ThreeVT

rename Cervicalspine Cspine

rename Thoracicspine Tspine

rename Lumbarspine Lspine

rename Sacralspine Sspine

rename Cordinsertion Cordinsert

rename BPDmm BPD

rename HCmm HC

rename ACmm AC

rename Femurmm FL

rename Nasalbonemm NBmm

rename Nuchalfoldmm NFmm

save "DATA Anatomy raw.dta", replace

use "DATA Anatomy raw.dta", clear

* next lines generate counters for each exam

* nSubDet is number of inadeqate view

* nAdqDet is number of adeequate views

* nAbnorm is number of abnormal views (a subset of adequate views)

* SubDetText is a running list of all the inaquate views

* Addtxt is the name of the view to add to SubDetText is view is inadquate

generate nSubDet = 0

generate nAdqDet=0

generate nAbnorm=0

generate SubDetTxt =""

generate Addtxt=""

global ITEM "Situs"

replace Addtxt="Situ "

replace Situs = "abnormal" if strpos(Situs,"inversus")>0

quietly do DO-FILE-itemrecode.do

global ITEM="LatVents"

replace Addtxt="LatV"

quietly do DO-FILE-itemrecode.do

global ITEM="Choroid"

replace Addtxt="Chrd"

quietly do DO-FILE-itemrecode.do

global ITEM="Falx"

replace Addtxt="Falx"

quietly do DO-FILE-itemrecode.do

global ITEM="Parenchyma"

replace Addtxt="Prnc"

quietly do DO-FILE-itemrecode.do

global ITEM="Cerebellum"

replace Addtxt="Cbel"

quietly do DO-FILE-itemrecode.do

global ITEM="Vermis"

replace Addtxt="Verm"

quietly do DO-FILE-itemrecode.do

global ITEM="Cisterna"

replace Addtxt="Cist"

quietly do DO-FILE-itemrecode.do

global ITEM="Cavum"

replace Addtxt="Cavm"

quietly do DO-FILE-itemrecode.do

global ITEM="Cranium"

replace Addtxt="Crnm"

quietly do DO-FILE-itemrecode.do

global ITEM="Profile"

replace Addtxt="Prof"

quietly do DO-FILE-itemrecode.do

global ITEM="Lips"

replace Addtxt="Lips"

quietly do DO-FILE-itemrecode.do

global ITEM="Nose"

replace Addtxt="Nose"

quietly do DO-FILE-itemrecode.do

global ITEM="Maxilla"

replace Addtxt="Mxla"

quietly do DO-FILE-itemrecode.do

global ITEM="Mandible"

replace Addtxt="Mand"

quietly do DO-FILE-itemrecode.do

global ITEM="Neck"

replace Addtxt="Neck"

quietly do DO-FILE-itemrecode.do

global ITEM="Diaphragm"

replace Addtxt="Dphr"

quietly do DO-FILE-itemrecode.do

*** Lungs recorded in Viewpoint as Rt and Lf separately.

*** The next several lines combine them into a single variable

global ITEM="Lung"

replace Addtxt="Lungs"

replace Rtlung="abnormal" if trim(Rtlung)=="Soft Marker Seen" | trim(Rtlung)=="details"

replace Rtlung="suboptimal" if Rtlung=="" | trim(Rtlung)=="not visualized"

replace Rtlung="normal" if trim(Rtlung)=="visualized"| strpos(Rtlung,"previously")>0

replace Ltlung="abnormal" if trim(Ltlung)=="Soft Marker Seen" | trim(Ltlung)=="details"

replace Ltlung="suboptimal" if Ltlung=="" | trim(Ltlung)=="not visualized"

replace Ltlung="normal" if trim(Ltlung)=="visualized"| strpos(Ltlung,"previously")>0

generate Lungs="suboptimal"

replace Lungs="normal" if Ltlung=="normal" & Rtlung=="normal"

replace Lungs="abnormal" if Ltlung=="abnormal" | Rtlung=="abnormal"

quietly do DO-FILE-itemrecode.do

global ITEM="Hands"

replace Addtxt="Hnds"

*** Hands recorded in Viewpoint as Rt and Lf separately.

*** The next several lines combine them into a single variable

replace Rthand="abnormal" if trim(Rthand)=="Soft Marker Seen" | trim(Rthand)=="details"

replace Rthand="suboptimal" if Rthand=="" | trim(Rthand)=="not visualized"

replace Rthand="normal" if trim(Rthand)=="visualized"| strpos(Rthand,"previously")>0

replace Lthand="abnormal" if trim(Lthand)=="Soft Marker Seen" | trim(Lthand)=="details"

replace Lthand="suboptimal" if Lthand=="" | trim(Lthand)=="not visualized"

replace Lthand="normal" if trim(Lthand)=="visualized"| strpos(Lthand,"previously")>0

generate Hands="suboptimal"

replace Hands="normal" if Lthand=="normal" & Rthand=="normal"

replace Hands="abnormal" if Lthand=="abnormal" | Rthand=="abnormal"

quietly do DO-FILE-itemrecode.do

global ITEM="Feet"

replace Addtxt="Feet"

*** Feet recorded in Viewpoint as Rt and Lf separately.

*** The next several lines combine them into a single variable

replace Rtfoot="abnormal" if trim(Rtfoot)=="Soft Marker Seen" | trim(Rtfoot)=="details"

replace Rtfoot="suboptimal" if Rtfoot=="" | trim(Rtfoot)=="not visualized"

replace Rtfoot="normal" if trim(Rtfoot)=="visualized"| strpos(Rtfoot,"previously")>0

replace Ltfoot="abnormal" if trim(Ltfoot)=="Soft Marker Seen" | trim(Ltfoot)=="details"

replace Ltfoot="suboptimal" if Ltfoot=="" | trim(Ltfoot)=="not visualized"

replace Ltfoot="normal" if trim(Ltfoot)=="visualized"| strpos(Ltfoot,"previously")>0

generate Feet="suboptimal"

replace Feet="normal" if Ltfoot=="normal" & Rtfoot=="normal"

replace Feet="abnormal" if Ltfoot=="abnormal" | Rtfoot=="abnormal"

quietly do DO-FILE-itemrecode.do

global ITEM="Stomach"

replace Addtxt="Stom"

quietly do DO-FILE-itemrecode.do

global ITEM "Kidneys"

replace Addtxt="Kids"

quietly do DO-FILE-itemrecode.do

global ITEM="Bladder"

replace Addtxt="Blad"

quietly do DO-FILE-itemrecode.do

global ITEM="Cordvessels"

replace Addtxt="3-VC"

*** Cord vessels is free text.

*** Next several lines reduce number of options

replace Cordvessels=lower(Cordvessels)

replace Cordvessels="suboptimal" if strpos(Cordvessels,"subop")>0 ///

| strpos(Cordvessels,"s/o")>0 ///

| strpos(Cordvessels,"not")>0 ///

| strpos(Cordvessels,"nws")>0

replace Cordvessels="abnormal" if strpos(Cordvessels,"2 vessel")>0 ///

| strpos(Cordvessels,"2vc") >0 ///

| strpos(Cordvessels,"single umbilical art") >0

replace Cordvessels="normal" if strpos(Cordvessels, "3 vessel")>0 ///

| strpos(Cordvessels, "3vc") >0 ///

| (strpos(Cordvessels, "normal") >0 & strpos(Cordvessels,"abnormal")==0)

quietly do DO-FILE-itemrecode.do

global ITEM="Cordinsert"

replace Addtxt="C-In"

quietly do DO-FILE-itemrecode.do

global ITEM="FourChamber"

replace Addtxt="4CH"

quietly do DO-FILE-itemrecode.do

global ITEM="RVOT"

replace Addtxt="RVOT"

quietly do DO-FILE-itemrecode.do

global ITEM="LVOT"

replace Addtxt="LVOT"

quietly do DO-FILE-itemrecode.do

global ITEM="ThreeVV"

replace Addtxt="3VV"

quietly do DO-FILE-itemrecode.do

global ITEM="ThreeVT"

replace Addtxt="3VT"

quietly do DO-FILE-itemrecode.do

global ITEM="AoArch"

replace Addtxt="Arch"

quietly do DO-FILE-itemrecode.do

global ITEM="Bicaval"

replace Addtxt="Bcvl"

*** BICAVAL COMBINED WITH SVC and IVC

generate Bicaval=""

replace Bicaval="normal" if trim(SVC)=="normal" & trim(IVC)=="normal"

replace Bicaval="suboptimal" if trim(SVC)=="suboptimal" | trim(IVC)=="suboptimal"

replace Bicaval="abnormal" if trim(SVC)=="abnormal" | trim(IVC)=="abnormal" ///

| trim(SVC)=="details" | trim(IVC)=="details"

quietly do DO-FILE-itemrecode.do

global ITEM "Cspine"

replace Addtxt="Cspn"

quietly do DO-FILE-itemrecode.do

global ITEM "Tspine"

replace Addtxt="Tspn"

quietly do DO-FILE-itemrecode.do

global ITEM "Lspine"

replace Addtxt="Lspn"

quietly do DO-FILE-itemrecode.do

global ITEM "Sspine"

replace Addtxt="Sspn"

quietly do DO-FILE-itemrecode.do

**** BIOMETRY

generate HCtext="measured"

replace HCtext="not meas" if BPD==. & HC==.

tabulate HCtext

generate ACtext="measured"

replace ACtext="not meas" if AC==.

tabulate ACtext

generate FLtext="measured"

replace FLtext="not meas" if FL==.

tabulate FLtext

generate NBtext="measured"

replace NBtext="not meas" if NBmm==.

replace NBtext="Under 15w" if GA<15

replace NBtext="Over 22w" if GA>22 & GA<.

tabulate NBtext

generate NFtext="measured"

replace NFtext="not meas" if NFmm==.

replace NFtext="Under 16w" if GA<16

replace NFtext="Over 20w" if GA>20 & GA<.

tabulate NFtext

save "DATA Anatomy recoded.dta", replace

use "DATA Anatomy recoded.dta", clear

generate tGA = trunc(GA)

* tGA is truncated GA (ie, completed weeks of gestation)

generate Incomplete=1

replace Incomplete=0 if nSubDet==0

generate Anyabnorm=0

replace Anyabnorm=1 if nAbnorm>0 & nAbnorm<.

**** REMAINDER OF ANALYSIS RESTRICTED TO 18w0d to 23w6d****

keep if GA>=18 & GA<24

save temp1823.dta, replace

*** (Execpt full GA range called up later for Figures)

*** For TABLE 1 inadequate views

tabulate Situs

tabulate LatVents

tabulate Choroid

tabulate Falx

tabulate Parenchyma

tabulate Cerebellum

tabulate Vermis

tabulate Cisterna

tabulate Cavum

tabulate Cranium

tabulate Profile

tabulate Lips

tabulate Nose

tabulate Maxilla

tabulate Mandible

tabulate Neck

tabulate Lungs

tabulate Diaphragm

tabulate Hands

tabulate Feet

tabulate Stomach

tabulate Kidneys

tabulate Bladder

tabulate Cordvessels

tabulate Cordinsert

tabulate FourChamber

tabulate RVOT

tabulate LVOT

tabulate AoArch

tabulate Bicaval

tabulate ThreeVV

tabulate ThreeVT

tabulate Cspine

tabulate Tspine

tabulate Lspine

tabulate Sspine

* TABLE 2 - Obesity, CS, 18 wks, AMA

generate AMA=0

replace AMA=1 if MatAge >=35

tabulate AMA Incomplete, row exact nokey

generate Wk18=0

replace Wk18=1 if GA<19

* variable Wk18 is 1 if GA is 18w0d to 18w6d

tabulate Wk18 Incomplete, row exact nokey

tabulate PriorCS Incomplete, row exact nokey

tabulate Obese Incomplete, row exact nokey

generate nObese=0

* variable nObese is Obesity recoded as a numeric variable (1=yes, 0=no) rather than a string variable

replace nObese=1 if trim(Obese)=="BMI 30-39.9"

replace nObese=1 if trim(Obese)=="BMI 40+"

tabulate nObese Incomplete, row exact nokey

logistic Incomplete nObese

logistic Incomplete PriorCS

logistic Incomplete Wk18

logistic Incomplete AMA

logistic Incomplete AMA Wk18 PriorCS nObese

**** FOR TABLE 3 - Whole-practice statistics

tabulate Incomplete

tabulate Anyabnorm

tabulate HCtext

tabulate ACtext

tabulate FLtext

* number of inadequate views

summarize nSubDet if nSubDet>0, detail

* summarize detail shows median, and 25th and 75th percentiles (for IQR)

* next line is for text in Section 3.3 - most common single inadequate views

tabulate SubDetTxt if nSubDet==1

*Nuchal Fold and Nose Bone at restricted GA

tabulate NFtext if GA<=20

tabulate NBtext if GA<=22

* next two lines are for text in Section 3.3 - missing measurements vs inadequate views

tabulate NFtext Neck if GA<=20, col exact

tabulate NBtext Profile if GA<=22, col exact

*** TABLE 4 BY SONOGRAPHER

tabulate Tech Incomplete, row nokey

* next two lines count how many exams by each sonographer & restricts tabulation to sonographers with at least 50 exams

bysort Tech: generate nExams=_N

tabulate Tech Incomplete if nExams >=50, row exact chi nokey

* next two lines find median and IQR of number of inadequate views, by tech and overall

bysort Tech: summarize nSubDet if nSubDet>0 & nExams >=50, detail

summarize nSubDet if nSubDet>0 & nExams >=50, detail

* next line is Kruskal-Wallis test on number of inadequate views.

kwallis nSubDet if nSubDet>0 & nExams >-50, by(Tech)

* next two lines show missing Nuchal Fold and Nasal Bone measurements

tabulate Tech NFtext if GA <=20 & nExams >=50, row chi nokey

tabulate Tech NBtext if GA <=22 & nExams >=50, row chi nokey

**** Significance tests for table 4

* You will need to CUSTOMIZE the names here.

* You can add analagous lines if you have additional sonographers to analyze

generate T1=0

generate T2=0

generate T3=0

generate T4=0

replace T1=1 if Tech=="A Anderson RDMS"

replace T2=1 if Tech=="B Bronson RDMS"

replace T3=1 if Tech=="D Donaldson RDMS"

replace T4=1 if Tech=="F Fredrickson RDMS"

* Next lines show incomplete exams per sonographer compared to rest of the practice, adjusted for Obesity, prior CS, AMA, GA 18 wks.

* In the tabulated results for sonographer T1, if "P >|z|" is <0.05, sonographer T1 is significantly different than rest of practice.

* If Odds ratio is >1, T1 has higher rate of incomplete exams. If odds ratio is <1, T1 has lower rate of incomplete exams.

logistic Incomplete T1 nObese PriorCS AMA Wk18

logistic Incomplete T2 nObese PriorCS AMA Wk18

logistic Incomplete T3 nObese PriorCS AMA Wk18

logistic Incomplete T4 nObese PriorCS AMA Wk18

* Next lines show rate of missing Nuchal Fold measurement compared to rest of the practice

* Fisher's exact <0.05 means that sonographer has signiicantly different rate than rest of the practice.

generate nNF=0

replace nNF=1 if trim(NFtext)=="measured"

tabulate T1 nNF if GA <=20, row nokey exact

tabulate T2 nNF if GA <=20, row nokey exact

tabulate T3 nNF if GA <=20, row nokey exact

tabulate T4 nNF if GA <=20, row nokey exact

* Next lines show rate of missing Nasal Bone measurement compared to rest of the practice

* Fisher's exact <0.05 means that sonographer has signiicantly different rate than rest of the practice.

generate nNB=0

replace nNB=1 if trim(NBtext)=="measured"

tabulate T1 nNB if GA <=22, row nokey exact

tabulate T2 nNB if GA <=22, row nokey exact

tabulate T3 nNB if GA <=22, row nokey exact

tabulate T4 nNB if GA <=22, row nokey exact

drop T1 T2 T3 T4 nExams nNF nNB

*** TABLE 5 - BY DOC

tabulate Doc Incomplete, row nokey

* next two lines count how many exams by each physician & restricts tabulation to physicians with at least 50 exams

bysort Doc: generate nExams=_N

tabulate Doc Incomplete if nExams >=50, row exact chi nokey

* next two lines find median and IQR of number of inadequate views, by Doc and overall

bysort Doc: summarize nSubDet if nSubDet>0 & nExams >=50, detail

summarize nSubDet if nSubDet>0 & nExams >=50, detail

* next line is Kruskal-Wallis test on number of inadequate views.

kwallis nSubDet if nSubDet>0 & nExams >-50, by(Doc)

* next two lines show missing Nuchal Fold and Nasal Bone measurements

tabulate Doc NFtext if GA <=20 & nExams >=50, row chi nokey

tabulate Doc NBtext if GA <=22 & nExams >=50, row chi nokey

**** Significance tests for table 4

* You will need to CUSTOMIZE the names here.

* You can add analagous lines if you have additional physicians to analyze

generate D1=0

generate D2=0

generate D3=0

generate D4=0

replace D1=1 if Doc=="J Johnson MD"

replace D2=1 if Doc=="M Markson DO"

replace D3=1 if Doc=="N Nicholson MD"

replace D4=1 if Doc=="P Pearson DO"

* Next lines show incomplete exams per physician compared to rest of the practice, adjusted for Obesity, prior CS, AMA, GA 18 wks.

* In the tabulated results for physician T1, if "P >|z|" is <0.05, physician T1 is significantly different than rest of practice.

* If Odds ratio is >1, T1 has higher rate of incomplete exams. If odds ratio is <1, T1 has lower rate of incomplete exams.

logistic Incomplete D1 nObese PriorCS AMA Wk18

logistic Incomplete D2 nObese PriorCS AMA Wk18

logistic Incomplete D3 nObese PriorCS AMA Wk18

logistic Incomplete D4 nObese PriorCS AMA Wk18

* Next lines show rate of missing Nuchal Fold measurement compared to rest of the practice

* Fisher's exact <0.05 means that physician has signiicantly different rate than rest of the practice.

generate nNF=0

replace nNF=1 if trim(NFtext)=="measured"

tabulate D1 nNF if GA <=20, row nokey exact

tabulate D2 nNF if GA <=20, row nokey exact

tabulate D3 nNF if GA <=20, row nokey exact

tabulate D4 nNF if GA <=20, row nokey exact

* Next lines show rate of missing Nasal Bone measurement compared to rest of the practice

* Fisher's exact <0.05 means that physician has signiicantly different rate than rest of the practice.

generate nNB=0

replace nNB=1 if trim(NBtext)=="measured"

tabulate D1 nNB if GA <=22, row nokey exact

tabulate D2 nNB if GA <=22, row nokey exact

tabulate D3 nNB if GA <=22, row nokey exact

tabulate D4 nNB if GA <=22, row nokey exact

drop D1 D2 D3 D4 nExams nNF nNB

*** For TABLE 6

* CUSTOMIZE Enter the name of whatever examiner you want to focus on.

*You can copy and paste these line to repeat them as many times as you need

use temp1823.dta, clear

keep if trim(Tech)=="A Anderson RDMS"

tabulate Situs

tabulate LatVents

tabulate Choroid

tabulate Falx

tabulate Parenchyma

tabulate Cerebellum

tabulate Vermis

tabulate Cisterna

tabulate Cavum

tabulate Cranium

tabulate Profile

tabulate Lips

tabulate Nose

tabulate Maxilla

tabulate Mandible

tabulate Neck

tabulate Lungs

tabulate Diaphragm

tabulate Hands

tabulate Feet

tabulate Stomach

tabulate Kidneys

tabulate Bladder

tabulate Cordvessels

tabulate Cordinsert

tabulate FourChamber

tabulate RVOT

tabulate LVOT

tabulate AoArch

tabulate Bicaval

tabulate ThreeVV

tabulate ThreeVT

tabulate Cspine

tabulate Tspine

tabulate Lspine

tabulate Sspine

* CUSTOMIZE for any additional examiner you want to tabulate

use temp1823.dta, clear

keep if trim(Doc)=="M Markson DO"

tabulate Situs

tabulate LatVents

tabulate Choroid

tabulate Falx

tabulate Parenchyma

tabulate Cerebellum

tabulate Vermis

tabulate Cisterna

tabulate Cavum

tabulate Cranium

tabulate Profile

tabulate Lips

tabulate Nose

tabulate Maxilla

tabulate Mandible

tabulate Neck

tabulate Lungs

tabulate Diaphragm

tabulate Hands

tabulate Feet

tabulate Stomach

tabulate Kidneys

tabulate Bladder

tabulate Cordvessels

tabulate Cordinsert

tabulate FourChamber

tabulate RVOT

tabulate LVOT

tabulate AoArch

tabulate Bicaval

tabulate ThreeVV

tabulate ThreeVT

tabulate Cspine

tabulate Tspine

tabulate Lspine

tabulate Sspine

**************** FIGURES 1 to 4 ********************************

use "DATA Anatomy recoded.dta", clear

*** FIGURE 1 HISTOGRAM OF EXAM FREQUENCY

histogram GA, freq bins(26) ///

ytitle(Number of exams) ///

ylabel(,angle(0)) ///

xtitle("Gestational age, weeks")

graph save Graph "FIGURE 1 Histogram exams vs GA.gph", replace

*****FIGURE 2 - 6 panels INCOMPLETE BY GA BY STRUCTURE

generate tGA = trunc(GA)

* tGA is truncated GA (ie, completed weeks of gestation).

replace tGA=16 if tGA<16

replace tGA=35 if tGA>35 & tGA<.

** Panel 1 HEART VIEWS

* 4-chamber

generate iSub = 0

replace iSub=1 if trim(FourChamber)=="suboptimal" | trim(FourChamber)=="blank"

bysort tGA: egen s4CH=mean(iSub)

replace s4CH = 100 * s4CH

* RVOT

drop iSub

generate iSub = 0

replace iSub=1 if trim(RVOT)=="suboptimal" | trim(RVOT)=="blank"

bysort tGA: egen sRVOT=mean(iSub)

replace sRVOT = 100* sRVOT

* LVOT

drop iSub

generate iSub = 0

replace iSub=1 if trim(LVOT)=="suboptimal" | trim(LVOT)=="blank"

bysort tGA: egen sLVOT=mean(iSub)

replace sLVOT = 100* sLVOT

* AoArch

drop iSub

generate iSub = 0

replace iSub=1 if trim(AoArch)=="suboptimal" | trim(AoArch)=="blank"

bysort tGA: egen sAoArch=mean(iSub)

replace sAoArch = 100* sAoArch

* Bicaval

drop iSub

generate iSub = 0

replace iSub=1 if trim(Bicaval)=="suboptimal" | trim(Bicaval)=="blank"

bysort tGA: egen sBicaval=mean(iSub)

replace sBicaval = 100* sBicaval

* 3VV

drop iSub

generate iSub = 0

replace iSub=1 if trim(ThreeVV)=="suboptimal" | trim(ThreeVV)=="blank"

bysort tGA: egen s3VV=mean(iSub)

replace s3VV = 100* s3VV

* 3VT

drop iSub

generate iSub = 0

replace iSub=1 if trim(ThreeVT)=="suboptimal" | trim(ThreeVT)=="blank"

bysort tGA: egen sThreeVT=mean(iSub)

replace sThreeVT = 100* sThreeVT

* GRAPH

graph twoway line s4CH sRVOT sLVOT sAoArch sBicaval s3VV sThreeVT tGA, ///

title(Cardiac Views) ///

ytitle("Inadequate Views, percent") ylabel(, angle(0)) ///

ylabel(0 (10) 40) ///

xlabel(15 (5) 35) ///

xtitle("Gestational Age, weeks") ///

color(black navy orange green red blue purple) ///

lpattern(line shortdash_dot longdash_dot shortdash dash dash dash) ///

legend(position(12) ring(0) cols(1) ///

label(1 4-chamber) ///

label(2 RVOT) label(3 LVOT) ///

label(4 Aortic Arch) label(5 SVC & IVC) ///

label(6 3-vessel) label(7 3-vessel trachea))

graph save Graph "FIGURE 2 Heart Views.gph", replace

** PANEL 2 SPINE VIEWS

* Cspine

drop iSub

generate iSub = 0

replace iSub=1 if trim(Cspine)=="suboptimal" | trim(Cspine)=="blank"

bysort tGA: egen sCspine=mean(iSub)

replace sCspine = 100* sCspine

* Tspine

drop iSub

generate iSub = 0

replace iSub=1 if trim(Tspine)=="suboptimal" | trim(Tspine)=="blank"

bysort tGA: egen sTspine=mean(iSub)

replace sTspine = 100* sTspine

* Lspine

drop iSub

generate iSub = 0

replace iSub=1 if trim(Lspine)=="suboptimal" | trim(Lspine)=="blank"

bysort tGA: egen sLspine=mean(iSub)

replace sLspine = 100* sLspine

* Sspine

drop iSub

generate iSub = 0

replace iSub=1 if trim(Sspine)=="suboptimal" | trim(Sspine)=="blank"

bysort tGA: egen sSspine=mean(iSub)

replace sSspine = 100* sSspine

* GRAPH

graph twoway line sCspine sTspine sLspine sSspine tGA, ///

title(Spine Views) ///

ytitle("Inadequate, percent") ylabel(, angle(0)) ///

ylabel(0 (10) 40) ///

xlabel(15 (5) 35) ///

xtitle("Gestational Age, weeks") ///

color(black navy orange green) ///

lpattern(line shortdash_dot longdash_dot shortdash) ///

legend(position(12) ring(0) cols(1) ///

label(1 Cervical) ///

label(2 Thoracic) label(3 Lumbar) ///

label(4 Sacral))

graph save Graph "FIGURE 2 Spine Views.gph", replace

** PANEL 3 FACE VIEWS

* Profile

drop iSub

generate iSub = 0

replace iSub=1 if trim(Profile)=="suboptimal" | trim(Profile)=="blank"

bysort tGA: egen sProfile=mean(iSub)

replace sProfile = 100* sProfile

* Lips

drop iSub

generate iSub = 0

replace iSub=1 if trim(Lips)=="suboptimal" | trim(Lips)=="blank"

bysort tGA: egen sLips=mean(iSub)

replace sLips = 100* sLips

* Nose

drop iSub

generate iSub = 0

replace iSub=1 if trim(Nose)=="suboptimal" | trim(Nose)=="blank"

bysort tGA: egen sNose=mean(iSub)

replace sNose = 100* sNose

* Maxilla

drop iSub

generate iSub = 0

replace iSub=1 if trim(Maxilla)=="suboptimal" | trim(Maxilla)=="blank"

bysort tGA: egen sMaxilla=mean(iSub)

replace sMaxilla = 100* sMaxilla

* Mandible

drop iSub

generate iSub = 0

replace iSub=1 if trim(Mandible)=="suboptimal" | trim(Mandible)=="blank"

bysort tGA: egen sMandible=mean(iSub)

replace sMandible = 100* sMandible

* Neck

drop iSub

generate iSub = 0

replace iSub=1 if trim(Neck)=="suboptimal" | trim(Neck)=="blank"

bysort tGA: egen sNeck=mean(iSub)

replace sNeck = 100* sNeck

* GRAPH

graph twoway line sProfile sLips sNose sMaxilla sMandible sNeck tGA, ///

title(Face Views) ///

ytitle("Inadequate Views, percent") ylabel(, angle(0)) ///

ylabel(0 (10) 60) ///

xlabel(15 (5) 35) ///

xtitle("Gestational Age, weeks") ///

color(black navy orange green red blue) ///

lpattern(line shortdash_dot longdash_dot shortdash dash dash) ///

legend(position(12) ring(0) cols(1) ///

label(1 Profile) ///

label(2 Lip) label(3 Nose) ///

label(4 Mandible) label(5 Maxilla) label(6 Neck))

graph save Graph "FIGURE 2 Face Views.gph", replace

** PANEL 4 BRAIN VIEWS

* LatVents

drop iSub

generate iSub = 0

replace iSub=1 if trim(LatVents)=="suboptimal" | trim(LatVents)=="blank"

bysort tGA: egen sLatVents=mean(iSub)

replace sLatVents = 100* sLatVents

* Falx

drop iSub

generate iSub = 0

replace iSub=1 if trim(Falx)=="suboptimal" | trim(Falx)=="blank"

bysort tGA: egen sFalx=mean(iSub)

replace sFalx = 100* sFalx

* Choroid

drop iSub

generate iSub = 0

replace iSub=1 if trim(Choroid)=="suboptimal" | trim(Choroid)=="blank"

bysort tGA: egen sChoroid=mean(iSub)

replace sChoroid = 100* sChoroid

* Cerebellum

drop iSub

generate iSub = 0

replace iSub=1 if trim(Cerebellum)=="suboptimal" | trim(Cerebellum)=="blank"

bysort tGA: egen sCerebellum=mean(iSub)

replace sCerebellum = 100* sCerebellum

* Vermis

drop iSub

generate iSub = 0

replace iSub=1 if trim(Vermis)=="suboptimal" | trim(Vermis)=="blank"

bysort tGA: egen sVermis=mean(iSub)

replace sVermis = 100* sVermis

* Cavum

drop iSub

generate iSub = 0

replace iSub=1 if trim(Cavum)=="suboptimal" | trim(Cavum)=="blank"

bysort tGA: egen sCavum=mean(iSub)

replace sCavum = 100* sCavum

* Cisterna

drop iSub

generate iSub = 0

replace iSub=1 if trim(Cisterna)=="suboptimal" | trim(Cisterna)=="blank"

bysort tGA: egen sCisterna=mean(iSub)

replace sCisterna = 100* sCisterna

* GRAPH

graph twoway line sLatVents sChoroid sCerebellum sVermis sCisterna sCavum tGA, ///

title(Brain Views) ///

ytitle("Inadequate Views, percent") ylabel(, angle(0)) ///

ylabel(0 (10) 70) ///

xlabel(15 (5) 35) ///

xtitle("Gestational Age, weeks") ///

color(black navy orange green red) ///

lpattern(line shortdash_dot longdash_dot shortdash dash) ///

legend(position(12) ring(0) cols(1) ///

label(1 Lateral ventricles) ///

label(2 Choroid plexus) label(3 Cerebellum) ///

label(4 Cerebellar vermis) label(5 Cisterna magna) label(6 Cavum septi pellucidi))

graph save Graph "FIGURE 2 Brain Views.gph", replace

** PANEL 5 ABDOMEN & CORD VIEWS

* Stomach

drop iSub

generate iSub = 0

replace iSub=1 if trim(Stomach)=="suboptimal" | trim(Stomach)=="blank"

bysort tGA: egen sStomach=mean(iSub)

replace sStomach = 100* sStomach

* Kidneys

drop iSub

generate iSub = 0

replace iSub=1 if trim(Kidneys)=="suboptimal" | trim(Kidneys)=="blank"

bysort tGA: egen sKidneys=mean(iSub)

replace sKidneys = 100* sKidneys

* Bladder

drop iSub

generate iSub = 0

replace iSub=1 if trim(Bladder)=="suboptimal" | trim(Bladder)=="blank"

bysort tGA: egen sBladder=mean(iSub)

replace sBladder = 100* sBladder

* Cordvessels

drop iSub

generate iSub = 0

replace iSub=1 if trim(Cordvessels)=="suboptimal" | trim(Cordvessels)=="blank"

bysort tGA: egen sCordvessels=mean(iSub)

replace sCordvessels = 100* sCordvessels

* Cordinsert

drop iSub

generate iSub = 0

replace iSub=1 if trim(Cordinsert)=="suboptimal" | trim(Cordinsert)=="blank"

bysort tGA: egen sCordinsert=mean(iSub)

replace sCordinsert = 100* sCordinsert

* GRAPH

graph twoway line sStomach sKidneys sBladder sCordvessels sCordinsert tGA, ///

title(Abdomen & Cord Views) ///

ytitle("Inadequate Views, percent") ylabel(, angle(0)) ///

ylabel(0 (10) 70) ///

xlabel(15 (5) 35) ///

xtitle("Gestational Age, weeks") ///

color(black navy orange green red) ///

lpattern(line shortdash_dot longdash_dot shortdash dash) ///

legend(position(12) ring(0) cols(1) ///

label(1 Stomach) ///

label(2 Kidneys) label(3 Bladder) ///

label(4 Cord vessel number) label(5 Cord insertion) )

graph save Graph "FIGURE 2 Abd-Cord Views.gph", replace

** PANEL 6 MISC VIEWS

* Situs

drop iSub

generate iSub = 0

replace iSub=1 if trim(Situs)=="suboptimal" | trim(Situs)=="blank"

bysort tGA: egen sSitus=mean(iSub)

replace sSitus = 100* sSitus

* Lungs

drop iSub

generate iSub = 0

replace iSub=1 if trim(Lungs)=="suboptimal" | trim(Lungs)=="blank"

bysort tGA: egen sLungs=mean(iSub)

replace sLungs = 100* sLungs

* Diaphragm

drop iSub

generate iSub = 0

replace iSub=1 if trim(Diaphragm)=="suboptimal" | trim(Diaphragm)=="blank"

bysort tGA: egen sDiaphragm=mean(iSub)

replace sDiaphragm = 100* sDiaphragm

* Hands

drop iSub

generate iSub = 0

replace iSub=1 if trim(Hands)=="suboptimal" | trim(Hands)=="blank"

bysort tGA: egen sHands=mean(iSub)

replace sHands = 100* sHands

* Feet

drop iSub

generate iSub = 0

replace iSub=1 if trim(Feet)=="suboptimal" | trim(Feet)=="blank"

bysort tGA: egen sFeet=mean(iSub)

replace sFeet = 100* sFeet

* GRAPH

graph twoway line sSitus sLungs sDiaphragm sHands sFeet tGA, ///

title(Other Views) ///

ytitle("Inadequate Views, percent") ylabel(, angle(0)) ///

ylabel(0 (10) 70) ///

xlabel(15 (5) 35) ///

xtitle("Gestational Age, weeks") ///

color(black navy orange green red) ///

lpattern(line shortdash_dot longdash_dot shortdash dash) ///

legend(position(12) ring(0) cols(1) ///

label(1 Situs) ///

label(2 Lungs) label(3 Diaphragm) ///

label(4 Hands) label(5 Feet))

graph save Graph "FIGURE 2 Misc Views.gph", replace

*** FIGURE 3 INCOMPLETE BY GA BY OBESE

use temp1823.dta, clear

generate iSub=.

replace iSub=0 if trim(Obese)=="BMI <30"

replace iSub=1 if Incomplete==1 & trim(Obese)=="BMI <30"

bysort tGA: egen sNotObese = mean(iSub)

replace sNotObese=100*sNotObese

replace iSub=.

replace iSub=0 if strpos(Obese,"30-")>0

replace iSub=1 if strpos(Obese,"30-")>0 & Incomplete==1

bysort tGA: egen sBMI30=mean(iSub)

replace sBMI30=sBMI30*100

replace iSub=.

replace iSub=0 if strpos(Obese,"40")>0

replace iSub=1 if strpos(Obese,"40")>0 & Incomplete

bysort tGA: egen sBMI40=mean(iSub)

replace sBMI40=sBMI40*100

sort GA

graph twoway line sNotObese sBMI30 sBMI40 tGA, ///

ytitle("Incomplete Exams, percent") ylabel(, angle(0)) ///

ylabel(0 (20) 100) ///

xlabel(18 (1) 24) ///

xtitle("Gestational Age, weeks") ///

color(black blue red) ///

lpattern(line shortdash longdash) ///

legend(position(1) ring(0) cols(1) ///

label(1 "BMI <30") ///

label(2 "BMI 30-39.9") label(3 "BMI ≥40"))

graph save Graph "FIGURE 3 Complete by GA by Obese.gph", replace

tabulate tGA Incomplete, row nokey

bysort Obese: tabulate tGA Incomplete, row nokey

**** FIGURE 4 - HISTOGRAM OF NUMBER OF INADEQATE VIEWS

histogram nSubDet if nSubDet>0 & nSubDet<=20, ///

freq ///

xtitle(Number of Inadequate Views) ///

ytitle(Number of Exams) ///

ylabel(,angle(0)) ///

note("GA 18.0 to 23.9 weeks")

graph save Graph "FIGURE 4 Histogram Inadequate views.gph", replace

log close

exit

**DO-FILE-itemrecode.do**

*** Subroutine for recoding each item of anatomy

** You may need to CUSTOMIZE this if your anatomy items have different text choices

replace $ITEM="abnormal" if strpos($ITEM,"Soft")>0

replace $ITEM="abnormal" if trim($ITEM)=="details"

replace $ITEM="normal" if strpos($ITEM,"previously")>0

replace $ITEM="normal" if strpos($ITEM,"normal") >0 & strpos($ITEM,"abnormal")==0

replace $ITEM="normal" if trim($ITEM)=="visualized"

replace $ITEM="inadequate" if strpos($ITEM, "suboptimal") >0

replace $ITEM="inadequate" if trim($ITEM)=="not examined"

replace $ITEM="inadequate" if trim($ITEM)=="not visualized"

replace $ITEM="inadequate" if trim($ITEM)==""

replace SubDetTxt=SubDetTxt + Addtxt + " " if ($ITEM=="inadequate")

replace nSubDet=nSubDet+1 if $ITEM =="inadequate"

replace nAdqDet=nAdqDet+1 if $ITEM !="inadequate"

replace nAbnorm=nAbnorm+1 if $ITEM == "abnormal"

exit
