# Supplementary material for: Quantitative Approach to Quality Review of Prenatal Ultrasound Examinations: Incomplete Detailed Fetal Anatomy Exams
Source: J Clin Med. 2025 May 12;14(10):3356. doi: 10.3390/jcm14103356 (PMC12112216; doi:10.3390/jcm14103356)
Supplement: Supplementary file 1 [file jcm-14-03356-s001.zip › Supplementary files/Supplementary File 5 - Physician Workload.pdf]

Supplementary File 5
Associations Between Physician Workload, Incomplete Exams, and Abnormal Findings

Background: One of the peer reviewers of the original submission of this article suggested that there might be associations between physician workload (as measured by relative value units, RVU) and the rate of incomplete exams or the rate of abnormal exams. For example, if physicians with high work RVU also had high rate of incomplete exams, it might suggest that high workload causes quality to suffer.

Method: We calculated the total work RVU generation per physician in the 7 practices combined for all obstetric ultrasound procedures during 2024 (using Current Procedural Terminology codes 76801 through 76828). Work RVU for each procedure code was obtained from the Centers for Medicare and Medicaid Services table, available at https://www.cms.gov/Regulations-and-Guidance/Regulations-and-Policies/QuarterlyProviderUpdates/downloads/cms1476p_4.pdf.

To obtain the mean work RVUs per day, we divided each provider's total work RVUs by the number of days on which the provider performed ultrasound services. Each physician's rate of incomplete detailed anatomy exams and abnormal detailed anatomy exams was calculated for exams from 18.0 to 23.9 weeks of gestation. We then performed regression and correlation analysis to measure the associations between each physician's percentage of incomplete exams, percentage of exams with abnormalities, and mean work RVUs per day. To minimize spurious percentages due to small numbers of exams, the analysis was restricted to the 41 physicians who performed at least 40 detailed anatomy exams at 18.0 to 23.9 weeks of gestation during the year (median 267 exams, interquartile range 148-604). This excluded 6 physicians who performed from 1 to 37 exams each (median 14 exams).

Results: As shown in the left panel of Figure S1, there was a negative correlation between the rate of incomplete detailed anatomy exams and physician work RVUs, r = 0.33, p = 0.034. As shown in the right panel, there was also a negative correlation between the rate of abnormal findings on detailed anatomy exams and physician work RVU's, r = 0.35, p = 0.023.

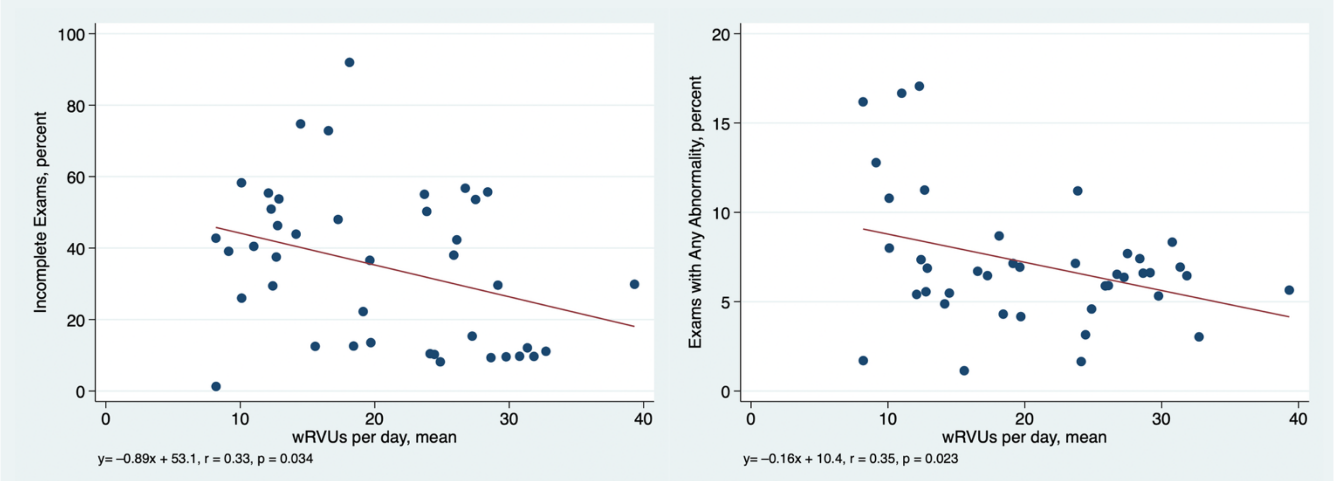

Figure S1. Correlations between incomplete detailed anatomy exams (left panel), abnormal exams (right panel), and physician workload expressed as mean work Relative Value Units (wRVUs) per day for obstetric ultrasound procedures. Each dot represents one physician. Red lines show linear regression fit. Regression equations shown in lower left corner of each panel.

As shown in Figure S2, there was a positive correlation between the rate of reported abnormalities on detailed anatomy exams and the rate of incomplete exams (r = 0.40, p = 0.009).

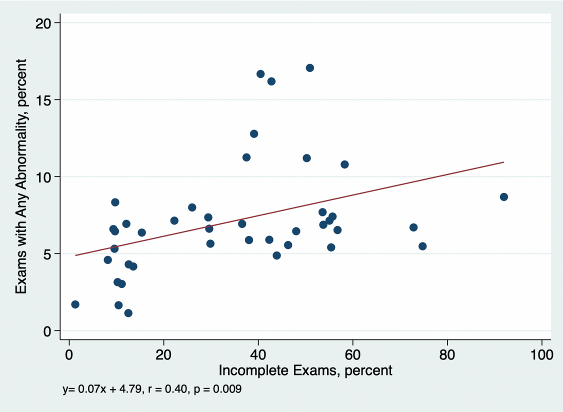

Figure S2. Correlations between the rate of abnormal detailed anatomy exams and the rate of incomplete exams. Each dot represents one physician. Red line shows linear regression fit. Regression equation shown in lower left corner.

Discussion: There are several possible explanations for these associations. For example, it may be that physicians with high daily work RVUs are more experienced and therefore are more capable of completing views that their sonographers are unable to complete. They may also be less likely interpret certain normal variant findings as abnormal. To evaluate this possibility, we would need to have more detailed information on each physician than is available in the Viewpoint database. Another possibility is that physicians with high daily work RVUs have more sonographers per day in their practice; with more personnel, it is possible that sonographers can spend extra time per exam to improve the rate of exam completion.

This exploratory study has several limitations. First, we based work RVUs only upon the ultrasound exams performed by each physician. MFM physicians typically also perform office visits and consultations, hospital visits and consultations, and procedures in both office and hospital. Because our study was restricted to data in the Viewpoint ultrasound reporting system, we had no way to capture the work RVUs for all these other activities, so therefore our total work RVU measurement expresses only a fraction of a physician's total workload. Second, we did not adjust the results to account for sonographer-level variation or practice-level variation in completion rates or abnormality rates. We also did not adjust for other known covariates such as obesity, prior cesarean, maternal age or gestational age 18.0-18.9 weeks. A complete analysis of all these factors would be beyond the scope of our current investigation. Future research in in this area may yield additional insights.

In conclusion, we found some statistically significant associations between physician ultrasound workload, rate of incomplete exams, and rate of abnormal exams. Within the limits of this first-level evaluation, it does not appear that high physician workload is associated with a high rate of incomplete exams.
